# Quantitative assessment of cell population diversity in single-cell landscapes

**DOI:** 10.1371/journal.pbio.2006687

**Published:** 2018-10-22

**Authors:** Qi Liu, Charles A. Herring, Quanhu Sheng, Jie Ping, Alan J. Simmons, Bob Chen, Amrita Banerjee, Wei Li, Guoqiang Gu, Robert J. Coffey, Yu Shyr, Ken S. Lau

**Affiliations:** 1 Department of Biostatistics, Vanderbilt University Medical Center, Nashville, Tennessee, United States of America; 2 Center for Quantitative Sciences, Vanderbilt University Medical Center, Nashville, Tennessee, United States of America; 3 Epithelial Biology Center, Vanderbilt University Medical Center, Nashville, Tennessee, United States of America; 4 Program in Chemical and Physical Biology, Vanderbilt University School of Medicine, Nashville, Tennessee, United States of America; 5 Department of Cell and Developmental Biology, Vanderbilt University School of Medicine, Nashville, Tennessee, United States of America; 6 Department of Medicine, Vanderbilt University Medical Center, Nashville, Tennessee, United States of America; 7 Veterans Affairs Medical Center, Tennessee Valley Healthcare System, Nashville, Tennessee, United States of America; Institute for Systems Biology, United States of America

## Abstract

Single-cell RNA sequencing (scRNA-seq) has become a powerful tool for the systematic investigation of cellular diversity. As a number of computational tools have been developed to identify and visualize cell populations within a single scRNA-seq dataset, there is a need for methods to quantitatively and statistically define proportional shifts in cell population structures across datasets, such as expansion or shrinkage or emergence or disappearance of cell populations. Here we present sc-UniFrac, a framework to statistically quantify compositional diversity in cell populations between single-cell transcriptome landscapes. sc-UniFrac enables sensitive and robust quantification in simulated and experimental datasets in terms of both population identity and quantity. We have demonstrated the utility of sc-UniFrac in multiple applications, including assessment of biological and technical replicates, classification of tissue phenotypes and regional specification, identification and definition of altered cell infiltrates in tumorigenesis, and benchmarking batch-correction tools. sc-UniFrac provides a framework for quantifying diversity or alterations in cell populations across conditions and has broad utility for gaining insight into tissue-level perturbations at the single-cell resolution.

## Author summary

Single-cell technologies generate hundreds to thousands of data points per sample, presenting a conundrum in determining similarities and differences across multiple samples. Currently, similarity is determined by the degree of “intermixing” of data points among samples, a local approach, but this approach cannot accurately evaluate the similarity of samples with cell populations close in data space but not overlapping. We present sc-UniFrac, an approach to compare hierarchical trees that represent single-cell landscapes, taking both global and local similarities into account. Furthermore, sc-UniFrac allows cells that drive differences between samples to be easily identified as unbalanced branches on trees. We used sc-UniFrac to evaluate experimental design based on biological and technical replicates, regional specification of brain cells, degree and identity of stromal infiltrate into tumor, and computational batch-correction tools. sc-UniFrac will be an important analysis tool going forward as the cost of single-cell technologies drops and more studies adopt multi-sample experimental designs.

## Introduction

Single-cell sequencing technologies enable profiling of hundreds to thousands of individual cells from a tissue composed of diverse cell types [1–8]. The rapid advances in single-cell technologies have led to a proliferation of novel computational tools. Current analyses mainly focus on identification of cell populations and transitional trajectories from data obtained from a single sample (reviewed in [9–12]). While these tools can shed light on complex biological processes within a given sample [13], it is becoming apparent that the power of single-cell technologies lies in multi-sample experiments, for which the response of single cells—in terms of identity and quantity following multiple perturbations—can be assessed. This poses a new statistical challenge: given that each sample is composed of transcriptomes from hundreds to thousands of individual cells, how does one compare across different samples to statistically assess population diversity and to detect population changes in an unbiased manner? As an increasing number of studies are generating single-cell RNA sequencing (scRNA-seq) data from multiple samples, there is an unmet need for a statistical framework that enables quantitative comparisons across different single-cell landscapes. Such a framework would have wide application from quantitatively uncovering cell population changes and assessing batch effect correction methods to classifying disease subtypes based upon single-cell landscapes.

There currently exists a paucity of approaches to determine cellular composition similarities and differences between samples. Citrus is a supervised approach for identifying cell populations that are significantly different between specified outcomes, and its goal is distinct from unsupervised comparison of samples based on similarities [14]. Another approach uses the Wasserstein metric known as Earth Mover’s Distance (EMD), which is a measure of the distance between two probability distributions over a certain data space [15]. Briefly, EMD partitions the entire data space into bins and measures the cost of transfer of data points from one distribution across these bins to resemble the other distribution. Orlova and colleagues applied EMD across datasets to quantify the similarity of two cell populations by measuring the distance between expression distributions in two-dimensional marker space [16]. However, partition of data spaces into bins has an exponential computational cost as the number of dimensions increases, limiting EMD use to one- or two-dimensional data spaces. Although there are multiple dimension-reduction approaches for high-dimensional data [11,17,18], analysis in a customizable, unrestricted number of dimensions would be preferred, especially for scRNA-seq datasets with thousands of native dimensions. We have developed the p-Creode score, which determines the similarities between p-Creode trajectories derived from a multidimensional single-cell landscape [19]. Most recently, cellAlign was developed to align single-cell trajectories using dynamic time warping, and an alignment-based distance was defined to evaluate similarities between trajectories [20]. However, these approaches are limited to datasets in which trajectories can be derived from continuous single-cell data and are not generalizable to all data distributions. Currently, the most common strategy for assessing similarities between single-cell landscapes remains a visual evaluation of the degree of “mixing” of data points when two or more samples are analyzed together on a t-distributed stochastic neighbor embedding (t-SNE) plot [2]. Two methods have recently been developed to use k-nearest neighbors to characterize this degree of intermixing [21,22]. Both methods use the simple assumption that k-nearest neighbors of each cell should have the same distribution of sample labels as the full dataset if the datasets are well mixed.

We were originally inspired by some of the early single-cell work in which similarities between replicates can be qualitatively evaluated by the degree of mixing of hierarchical clusters between replicates [7]. Thus, by deriving a quantitative measure to compare between hierarchical trees generated by clustering, we can obtain a corresponding quantitative, statistically testable metric to compare cell population diversity between single-cell landscapes. Multiple metrics to measure similarity between trees have been proposed, such as Baker’s gamma index [23], which we used previously to determine the similarity between signaling modules [24]. UniFrac is a distance metric originally devised to compute differences between microbial communities by incorporating phylogenetic information [25]. UniFrac provides a qualitative measure, which is calculated as the fraction of the total unshared branch lengths. A weighted version of UniFrac is a quantitative measure, such that branch lengths are weighted by the relative abundance of each taxon [26]. We created a workflow, called sc-UniFrac, that enables the application of the weighted UniFrac statistical framework on single-cell data to identify and characterize cellular diversity that distinguishes single-cell landscapes. Pairwise comparisons using sc-UniFrac can easily be extended to multi-sample experimental designs that are increasingly common in single-cell studies. sc-UniFrac compares diversity based on transcriptome similarities of single cells and is more powerful than intermixing methods based on its accounting of both the global and local structures of the data. We demonstrated the utility of sc-UniFrac in quantifying similarities between simulated and real sc-RNAseq datasets, for which the ground truth of similarities between samples is known. We also successfully applied sc-UniFrac to detect technical effects from replicate samples, assess the performance of batch-correcting methods, and implicate the sources of technical variation, although sc-UniFrac itself does not correct for these effects. We envision that quantitative metrics such as sc-UniFrac will find increasing utility as the field continues to generate a greater number of sc-RNAseq datasets from different conditions. sc-UniFrac will greatly facilitate single-cell studies, including those aimed at deciphering how cell populations respond to perturbations or tracking the evolution of cell populations during disease progression.

## Results

### Overview of sc-UniFrac

**Fig 1:**
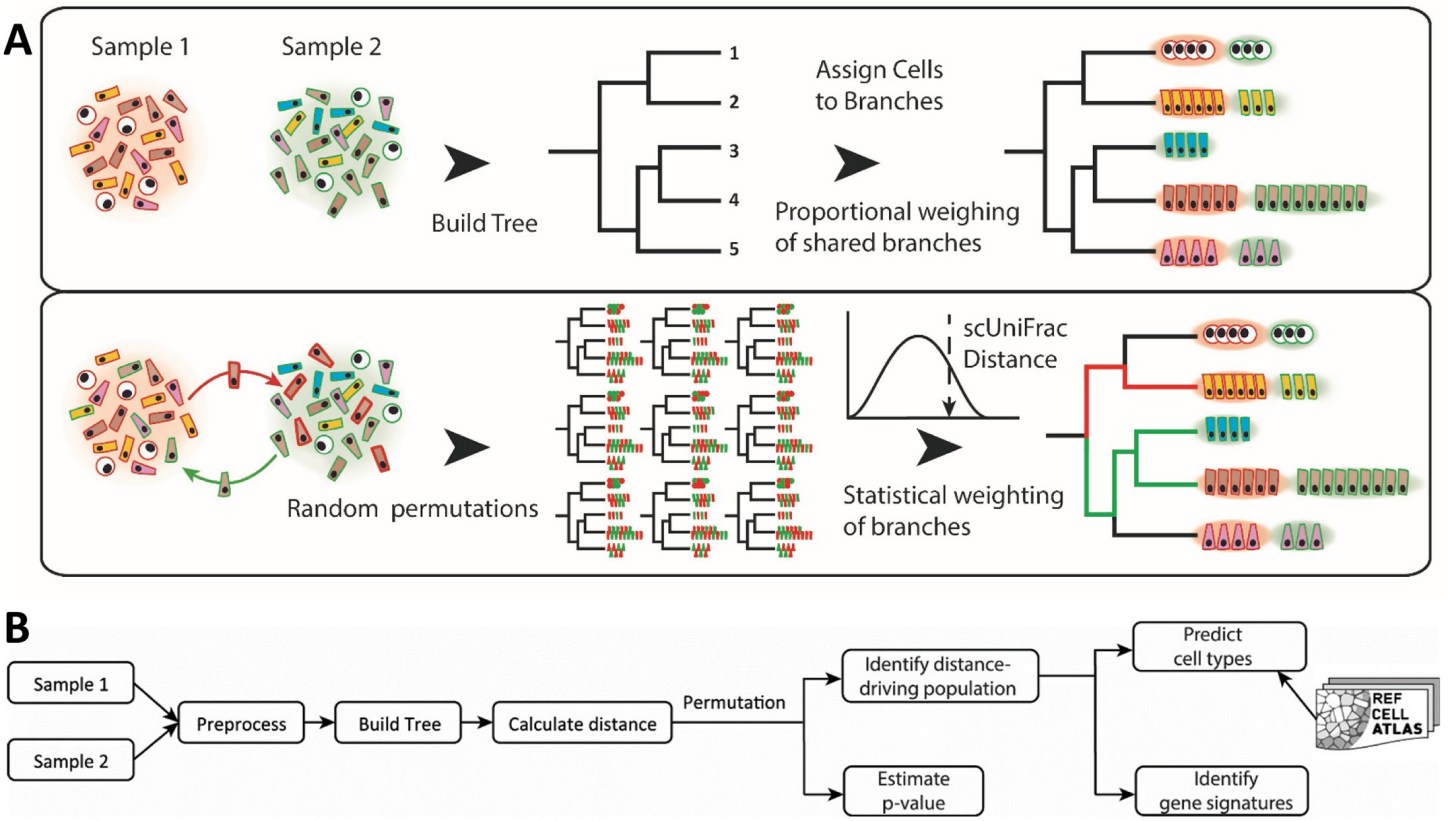
Overview of the sc-UniFrac method. (A) A hierarchical tree is built by clustering the combined single-cell transcriptome profiles from two samples and by calculating distances between cluster centroids. Each cell, as a function of their cluster membership, is then assigned to branches. Branch lengths weighted by the relative abundance of each sample are used to calculate the sc-UniFrac distance. In the second step, the sample labels of all cells are swapped without altering the tree topology to generate a null distribution of sc-UniFrac distances, where a *p*-value for the sc-UniFrac distance can be calculated. (B) Workflow overview of the sc-UniFrac package for characterizing dissimilarities between two samples.

For quantifying cell diversity differences in single-cell landscapes, we borrowed the UniFrac concept from the microbiome field. UniFrac is a distance metric for quantifying differences in phylogenetic diversity between ecological landscapes. Instead of operating on phylogenetic trees that describe microbial diversity between two samples, sc-UniFrac has been adopted for single-cell research and builds hierarchical trees from clustering analyte profiles (e.g., transcriptome) of single cells combined from two datasets. The purpose of clustering was not to separate cells into distinct groups, as performed traditionally for single-cell transcriptome datasets; instead, the hierarchical tree in sc-UniFrac is only to discern potential structures within the data. Thus, clustering can be performed with any method and can be applied to data with any distributions. The hierarchical tree can be constructed post hoc using distances between cluster centroids. The clustering tree, encompassing the structural features of cell subpopulations, is used for calculating the weighted UniFrac distance. sc-UniFrac is calculated by weighting relative abundance of samples assigned to each branch, as well as the branch length that denotes the distance between cluster centroids (Fig 1A). A permutation test, conducted by randomizing the sample labels of the cells without changing the tree topology, is used to calculate statistical significance of the overall sc-UniFrac distance (i.e., to test whether the cell populational structures between the two samples are identical) (see Methods). For single-cell data, it is very important to identify cell populations altered across conditions, which are derived from branches that have significant proportion shifts between two samples. Instead of relying on exact, nonzero proportional differences, which would find every branch and easily be skewed by outliers, sc-UniFrac leveraged the above permutation test to identify branches whose proportional shifts cannot occur by chance alone. This procedure corrects for the algorithm’s sensitivity to noisy outliers prevalent in scRNA-seq experiments. After identifying cell populations that drive the compositional difference between single-cell transcriptome landscapes, sc-UniFrac detects gene signatures that mark these cell populations. Finally, sc-UniFrac predicts the potential identities of these populations by matching individual cell signatures to cell types from reference atlases. sc-UniFrac can operate in two modes: (1) pairwise comparisons and (2) extension of the pairwise approach to a multi-sample experimental design. The general workflow of the sc-UniFrac pipeline is shown in Fig 1B.

Note that sc-UniFrac operates agnostically to technical or biological effects in the datasets and can be used to evaluate either depending on the input data. To evaluate technical effects, control replicate samples over multiple batches would be used as the input. To evaluate biological differences, it is assumed that technical variation in the input datasets has been well controlled for or batch correction has already been applied in previous processing steps.

### Sensitive and robust quantification of proportional shifts in single-cell population diversity

**Fig 2:**
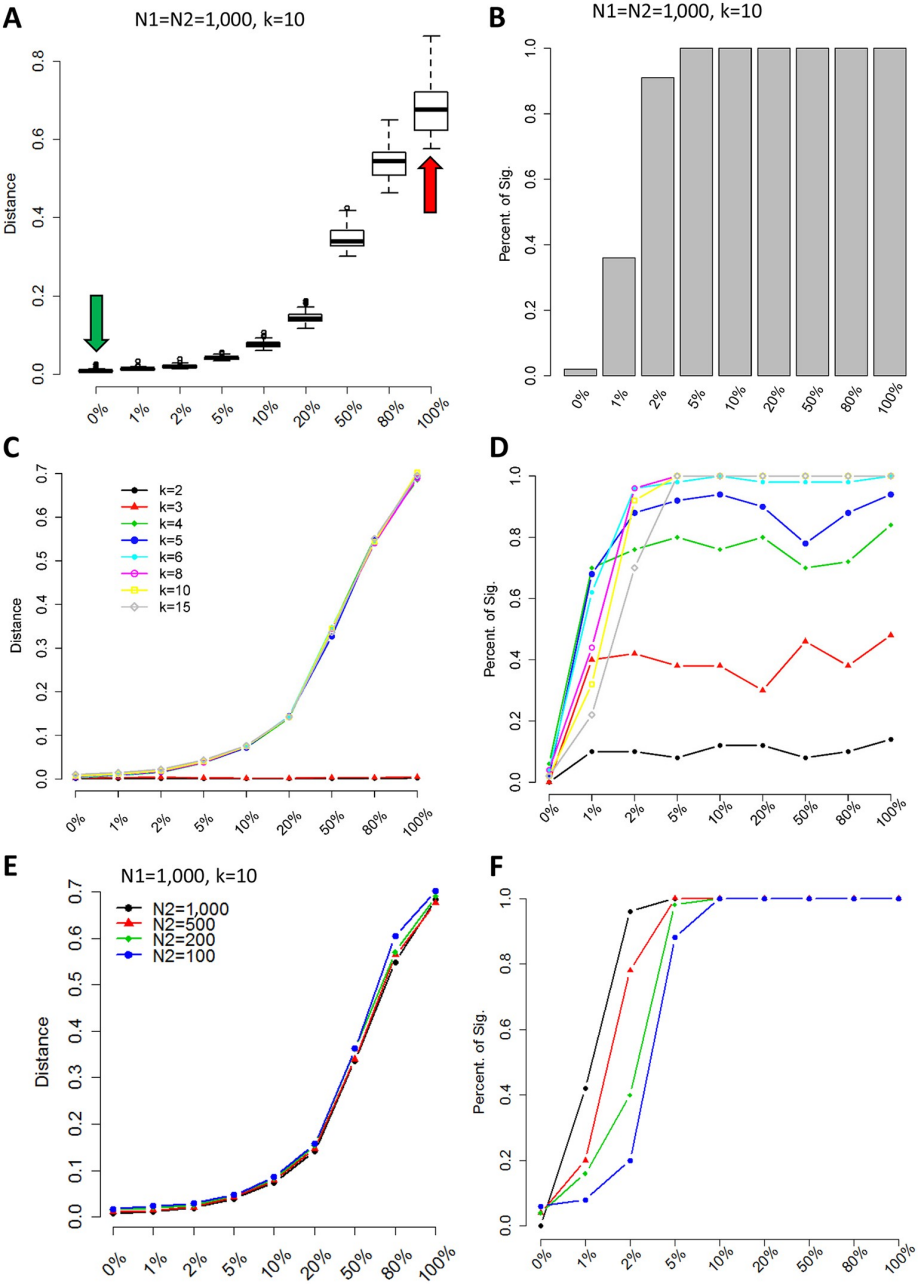
Simulation data reveal sc-UniFrac to be sensitive and robust. (A) Two groups (N1 and N2) of 1,000 cells were selected from CD8 and CD4 cells identified in the Wishbone dataset (S1 Data) [25]. N1 is always composed of 100% CD8 cells, while N2 is composed of CD8 cells and different proportions of CD4 cells (indicated on x-axis). Green and red arrows represent CD8/CD8 (completely similar) and CD8/CD4 (completely dissimilar) comparisons, respectively; y-axis is the sc-UniFrac distance calculated over *n* = 50 runs with k = 10. Boxes represent the first and third quartiles, and bars represent maximum and minimum values. (B) Sensitivity of sc-UniFrac evaluated by the fraction of incidences that a statistically significant sc-UniFrac distance was returned over *n* = 50 runs, as a function of increasing dissimilarity between N1 and N2 using the same simulation scheme as panel A. (C) Mean sc-UniFrac plotted as in panel A with varying k parameter. (D) Fraction significant sc-UniFrac detected plotted as in panel B with varying k parameter. (E) Mean sc-UniFrac plotted as in panel A with N1 = 1,000 but a varying N2 size to determine the effect of dataset size imbalance on sc-UniFrac. (F) Fraction significant sc-UniFrac detected plotted as in panel B with N1 = 1,000 and varying N2 size.

To evaluate the performance of sc-UniFrac in quantifying the compositional difference between single-cell landscapes, we first applied the method to experimental datasets in which population structure can be precisely controlled. Two 1,000-cell populations were generated by sampling from the CD8 and CD4 T-cell populations, respectively, from the T-cell development dataset of the mouse thymus (S1 Data) [25]. This process was repeated 50 times to evaluate the robustness of sc-UniFrac. Comparing the CD8 versus CD4 populations (1,000-cell sample from each population) using sc-UniFrac resulted in a large sc-UniFrac distance, indicating, as expected, completely different cell populations (Fig 2A—red arrow, S1A Fig). In contrast, comparing two 1,000-cell populations resampled from CD8 cells revealed that they possess the same population structures with a median distance of 0 (Fig 2A—green arrow, S1B Fig). We then evaluated the performance of sc-UniFrac in a simulation experiment in which we constructed a series of paired samples with a gradation of proportional shift in cell populations. For each pair, one sample included only CD8 cells (N1), while the other was composed of proportional mixtures of CD4 and CD8 cells (N2), starting from 0% of CD4 cells (no shift) to 100% of CD4 cells (complete shift). The distance of sc-UniFrac progressively increased as proportional shifts became larger (Fig 2A and S1C and S1D Fig). Among the 50 resampled runs, less than 0.2% of the sc-UniFrac distances generated were significantly different (identified as false positives) when two samples were identical (0% proportional shift), while over 95% of the distances were significantly different even when there was as little as 2% of CD4 cells mixed in with the CD8 cells (Fig 2B). These results signify that sc-UniFrac is sensitive and specific for detecting minute shifts in population structure.

Next, using this controlled sampling scheme, we evaluated the impact of changing various parameters on the performance of sc-UniFrac. First, we altered the k parameter for dividing the data into k subpopulations for analysis, which tunes the resolution by which sc-UniFrac analyzes the datasets. The quantitative ability and sensitivity of sc-UniFrac were robust as long as k was not exceedingly low (k > 3) (Fig 2C and 2D). At k ≤ 3, single-cell datasets are represented by three major groups or less, reducing the resolution of sc-UniFrac for detecting differences in single-cell level diversity. As k increases, the performance of sc-UniFrac continues to improve slowly until reaching an asymptote at the expense of higher computational costs. When k is extremely high, sc-UniFrac might be distracted by unimportant details (noise) and fail to capture global structures. Thus, we conclude that the performance of sc-UniFrac is minimally affected by the selection of k (as long as it is not too low or extremely high). We recommend a k in the range of 10 to 30 depending on the heterogeneity of the data.

Second, we assessed the effect of imbalanced dataset sizes. Keeping the size of sample 1 (N1) constant at 1,000 cells, we altered the size of sample 2 (N2) during resampling while maintaining the same population structure. The sc-UniFrac method was observed to be robust with respect to dataset size imbalances, with only minor losses in sensitivity at larger imbalances (detection limit of 5% instead of 2%) (Fig 2E and 2F and S2 Fig). These results demonstrate the robustness of sc-UniFrac, whose performance is independent of input parameters.

The same analysis using scRNA-seq data demonstrated similar results (S3 Fig). Here, we extracted erythrocyte and myeloid cell progenitor cell populations from the Paul and colleagues dataset (S2 and S3 Data) [26]. Using the erythrocyte as a base population (*N* = 500 cells), we progressively mixed in the progenitor cells to construct simulated datasets in a manner similar to the CD4/CD8 analysis. The general conclusions are the same, with two minor differences. First, this dataset is more robust to parameter changes due to the increased distinctiveness between the two cell populations. We speculate that better separation can be achieved either through true biological difference or by having more dimensions to define cell populations in scRNA-seq data compared with Cytometry Time-of-Flight (CyTOF) data. Second, sensitivity to detect dissimilarity is slightly decreased (from 2% to 5% disparate cells for k = 10, e.g.) due to smaller dataset sizes. Nevertheless, the robustness of the algorithm to the k parameter and dataset size imbalance still holds in this analysis.

### Assessment of reproducibility of scRNA-seq data and dissimilarities between different tissue samples

To demonstrate the utility of sc-UniFrac on scRNA-seq data, we generated a series of scRNA-seq datasets with known similarities and differences using inDrop (1Cell Bio; Cambridge, MA) sequencing [1]. These datasets included tissue samples from mouse colonic epithelium, consisting of both technical and biological replicates, as well as biological replicate samples of mouse embryonic pancreatic islets collected at embryonic day 14.5 (E14.5). Three technical replicates (colon1_1, colon1_2, colon1_3) consisted of multiple single-cell fractions collected from 1 mouse, whose libraries were constructed and sequenced on separate days. Three biological replicates (colon1, colon2, colon3) consisted of samples collected from different mice on different days but underwent library preparation and sequencing together. One sample (colon1) has both technical and biological replicates.

**Fig 3:**
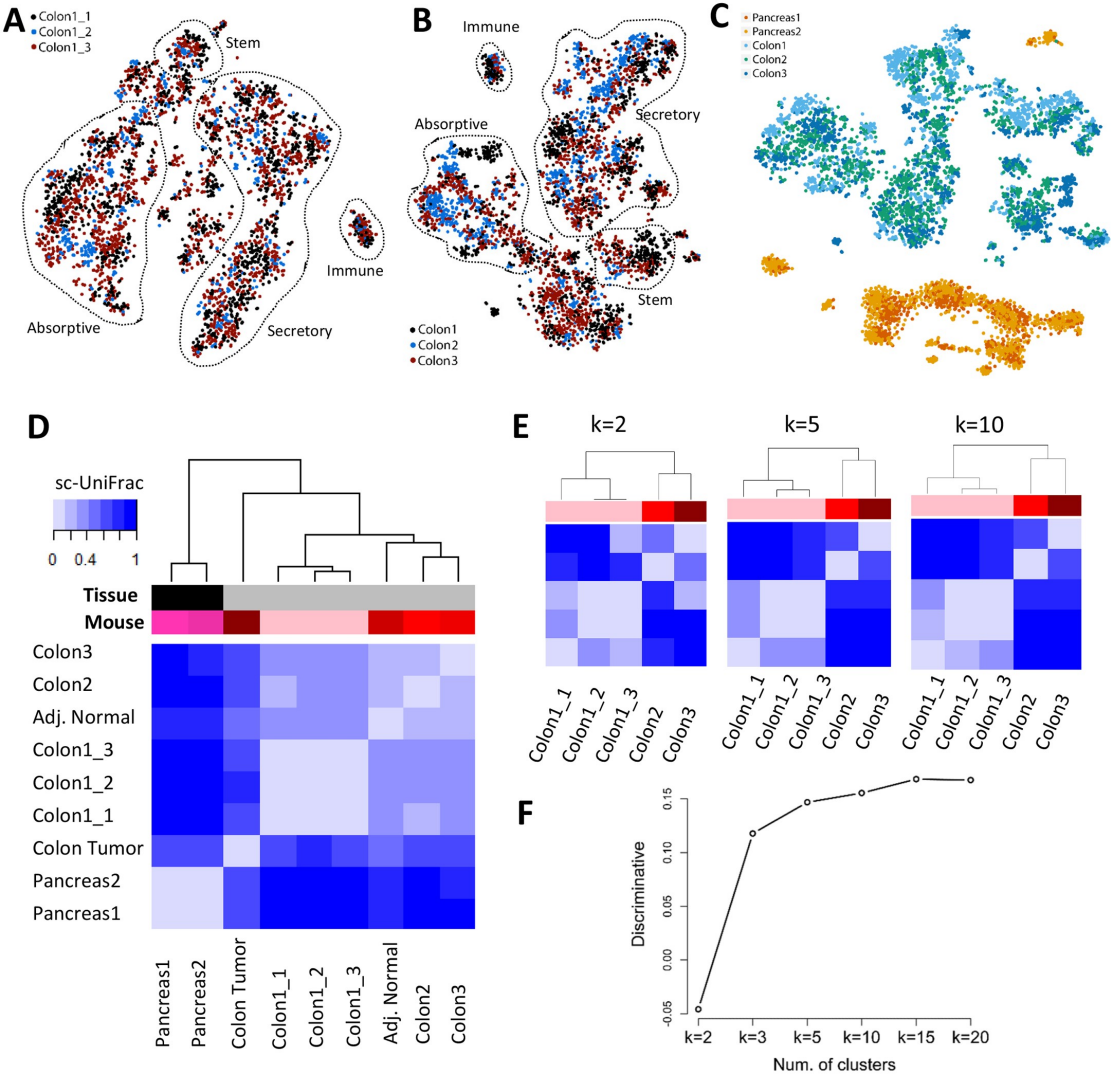
sc-UniFrac statistically determines dissimilarities between single-cell data landscapes. t-SNE plots of (A) technical and (B) biological replicates of scRNA-seq data generated from the adult murine colonic mucosa. Replicates were combined for t-SNE analyses and labeled with different colors. Outlined populations were identified with canonical markers. (C) t-SNE plot depicting E14.5 pancreatic islet and adult colonic mucosa scRNA-seq data in different mice, showing segregation by organ type. (D) Hierarchical clustering by sc-UniFrac of scRNA-seq landscapes generated from E14.5 pancreatic islet and adult colonic mucosa (indicated by tissue label), with technical and biological replicates (indicated by mouse label), as well as colonic tumor and adjacent normal isolated from an induced *Lrig1*^*CreERT2/+*^;*Apc*^*fl/+*^ mouse. Heat represents sc-UniFrac distance between two samples. (E) Hierarchical clustering by sc-UniFrac of single-cell landscapes of technical and biological replicates of the colonic mucosa while varying parameter k. (F) Discriminate analysis of sc-UniFrac on biological and technical replicates. Discriminative ability, as defined by the smallest distance between biological replicates minus the largest distance between technical replicates, plotted against k. Data from GSE102698, GSE114044, GSE117615, GSE117616. scRNA-seq, single-cell RNA-sequencing; t-SNE, t-distributed stochastic neighbor embedding.

Two traditional strategies to assess the reproducibility of scRNA-seq datasets—for both biological and technical replicates—were performed. One strategy is to compare the median levels of all genes expressed between every sample pair using Spearman correlation analysis. The correlation analysis demonstrated that the technical replicates were more similar among each other (mean R = 0.89 ± SD 0.04) compared with samples among biological replicates (mean R = 0.80 ± SD 0.07) (S4 Fig). As expected, the embryonic islets displayed a median gene expression that was the most different when compared to the adult colon (mean R = 0.504 ± SD 0.07). These results are consistent with the expected similarity among different conditions with technical replicates being more similar than biological replicates, which are more similar than the outgroup organ. While correlation analysis can quantify the degree of similarity in terms of the average transcriptional profile, it provides a very rough estimate and tends to be easily biased towards the dominant population in single-cell landscapes. The other strategy is to do a visual evaluation on how cells from multiple samples are intermixed on a t-SNE plot. Structural differences among different samples will be reflected in segregation of data points into separate clusters by sample, while data points from similar samples will appear together as mixed clusters. Visualization on a t-SNE plot showed the same result as median correlation analysis. Technical replicates appeared more intermixed within t-SNE clusters compared with biological replicates (Fig 3A and 3B and S5A and S5B Fig). In contrast, pancreatic islet biological replicates segregate away from samples generated from both the technical and biological replicates of the colon (Fig 3C). While t-SNE analysis describes the subpopulation structure of the samples, it is not quantitative in that similarities and differences were assessed subjectively by visualization.

Compared with these two traditional strategies, sc-UniFrac provides an objective, precise, and unbiased metric to quantify compositional dissimilarities across scRNA-seq datasets while taking population structures into account. The calculated sc-UniFrac distance between the colonic and pancreatic islet datasets was 1, the maximal obtainable distance demonstrating that the samples did not share any cell populations (Fig 3D and S5C Fig). Much smaller, but significant, distances were observed among biological replicates of colonic datasets (Fig 3D and S5C Fig, distance = 0.24–0.37), suggesting that they share cell populations but proportional compositional difference can still be detected. Technical replicates appeared the most similar with sc-UniFrac being marginally small without statistical significance (Fig 3D and S5C Fig, distance = 0.05–0.09), suggesting that they are composed of almost identical data points. The ordering by similarity across samples was robust to the k parameter (Fig 3E and 3F). A metric was defined to evaluate the power of sc-UniFrac for discriminating biological replicates from technical replicates (discriminative ability) by subtracting the smallest distance between biological replicates by the largest distance between technical replicates. A positive discriminative ability suggests that sc-UniFrac can discriminate technical from biological replicates. Notably, the k parameter again did not affect the discriminative ability except when k is very small (k ≤ 2) (Fig 3E and 3F). These results demonstrate the ability of sc-UniFrac to objectively and quantitatively determine dissimilarities between single-cell datasets, as seen by the ordering of samples by their expected similarities (technical replicate > biological replicate > outgroup organ).

**Fig 4:**
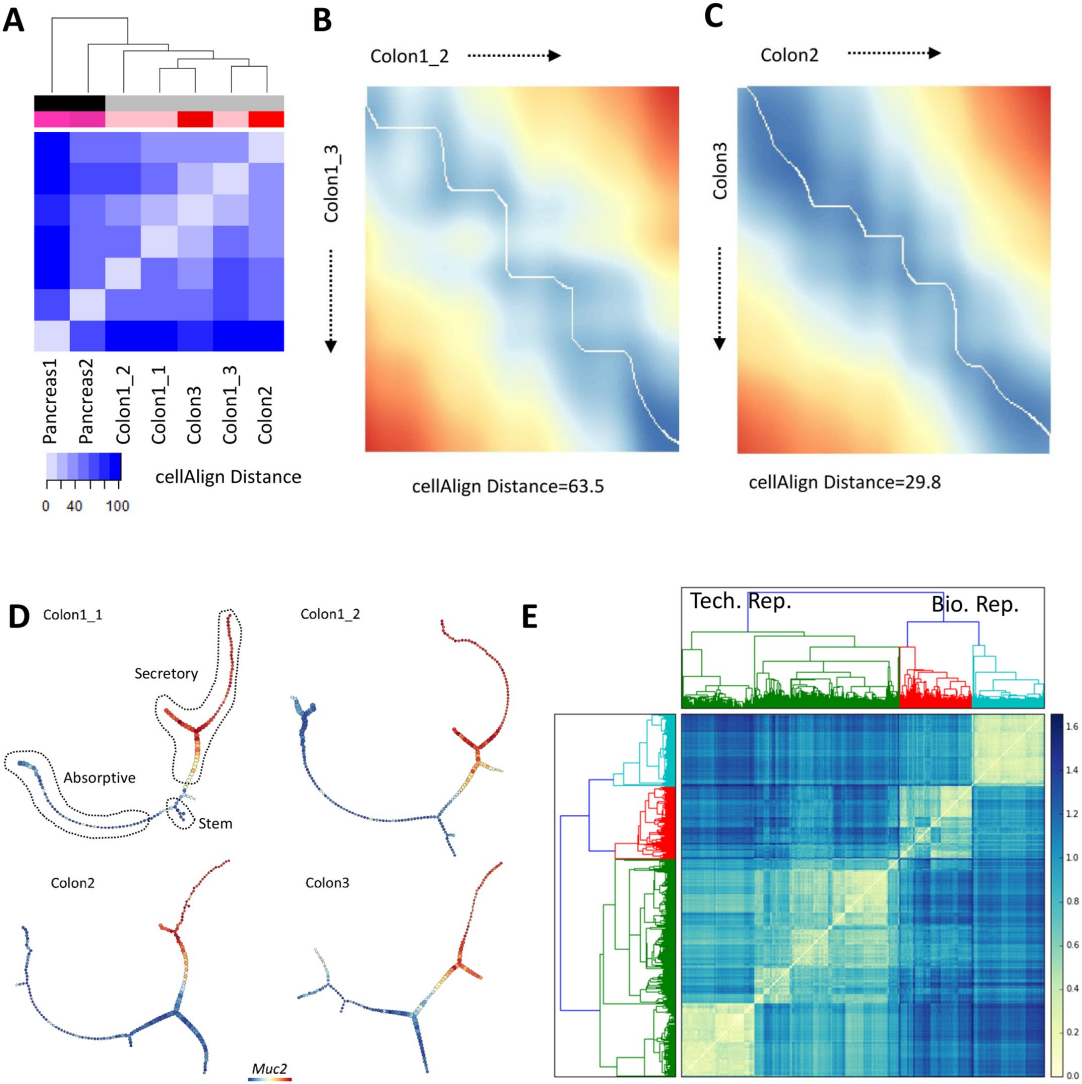
Alternative methods of landscape comparisons arrive at similar results compared with sc-UniFrac. (A) Hierarchical clustering by cellAlign distance calculated using unbranched trajectories created from scRNA-seq data generated from E14.5 pancreatic islet and adult colonic mucosa (indicated by tissue label-greyscale bar), with technical and biological replicates (indicated by mouse label-red bar) (S4 Data). Heat represents cellAlign distance between two samples. Example dissimilarity matrices resulting from alignments of unbranched stem cell to colonocyte trajectories using the cellAlign algorithm according to [20] for (B) technical replicates and (C) biological replicates. Normalized alignment-based distances appear below each matrix. (D) Representative p-Creode trajectories depicting the colonic epithelial differentiation continuum of 2 technical and 2 biological replicates. Outlined lineages were identified with canonical markers. *Muc2* expression overlay. (E) Hierarchical clustering by p-Creode scoring of trajectories generated from scRNA-seq data of technical (green) and biological (red, cyan) replicates. *N* = 100 resampled p-Creode runs for each dataset were performed and then analyzed together in a single clustering analysis. Heat represents the p-Creode score between two trajectories. Data from GSE102698, GSE114044, GSE117616; https://github.com/KenLauLab/pCreode_Comparison_Across_Datasets. scRNA-seq, single-cell RNA sequencing.

We performed a comparison of sc-UniFrac to published methods for assessing single-cell landscape similarity, using the above dataset with known similarity ordering. First, we evaluated cellAlign, an algorithm for aligning two unbranched pseudotemporal trajectories using dynamic time warping [20]. The alignment-based distance was defined to evaluate the similarity of two trajectories. cellAlign requires the input to be continuous scRNA-seq data comprising single unbranched trajectories. To generate such an input, we manually selected cells that form unbranched data continua from stem cells to colonocytes for the colonic datasets as well as from endocrine progenitors to beta cells for the pancreatic islet datasets (S4 Data). The cellAlign distance, similar to sc-UniFrac, revealed that colonic datasets can be distinguished and clustered away from pancreatic islet datasets, while biological and technical replicates of the colon cannot be clearly delineated (Fig 4A). An example of a nonideal alignment between technical replicates compared with an alignment between biological replicates is shown (Fig 4B and 4C). These results suggest that sc-UniFrac is more powerful for distinguishing differences between single-cell landscapes than cellAlign. The low sensitivity of cellAlign might be due to various parameters, such as dataset size imbalance or uneven sampling of datapoints along a trajectory, which we have not thoroughly tested here. Furthermore, the application of cellAlign is restricted to the very specific case of continuous data that form a single unbranched trajectory, thus limiting its generalizability.

To extend orthogonal methodologic comparisons to sc-UniFrac, we also used p-Creode trajectory analysis, an algorithm by which a single-cell landscape composed of continuous cell-state data is represented as acyclic graphs to model transition trajectories [19]. The p-Creode score, developed to determine the topological similarity between graphs with differing nodes and edges, can be used to quantify dissimilarities of the trajectory graph outputs generated from different datasets. We revised the p-Creode scoring method to accommodate comparisons of graphs of difference sizes by interpolating between edges connecting nodes instead of directly matching node positions between the two test datasets (S6A–S6C Fig and Methods). p-Creode was applied to each dataset for 100 times to generate consensus trajectories using data resampling. The modified p-Creode score was used as a distance metric for clustering cell-transitional trajectories created from the resampled datasets. Consistent with sc-UniFrac, p-Creode trajectories among technical replicates clustered together using the p-Creode score as a dissimilarity metric, while data from biological replicates were more disparate (Fig 4D and 4E). As expected, organ specificity drove clustering when pancreatic islet data were added to the analysis, with all trajectories generated from colonic data clustering together away from pancreatic trajectories (S6D and S7 Figs). Similar to cellAlign, p-Creode was designed for data that are distributed as a continuum, and not as distinct clusters. However, the p-Creode score can also be used to evaluate complex multi-branching trajectories in addition to linear ones. Nevertheless, comparisons can only be made for tissue systems that are transitioning, which is the case for both the adult colonic epithelium and embryonic pancreatic islets compared here. sc-UniFrac does not have this limitation because it can compare between datasets of any distribution, including continuous data, as well as discrete populations that are composed of cells from different lineages. Thus, sc-UniFrac has greater general utility than the above two trajectory comparison methods for determining dissimilarities of single-cell datasets in an unsupervised way without prior knowledge of the distribution of the data.

### Identification of cell populations that drive differences between single-cell landscapes

**Fig 5:**
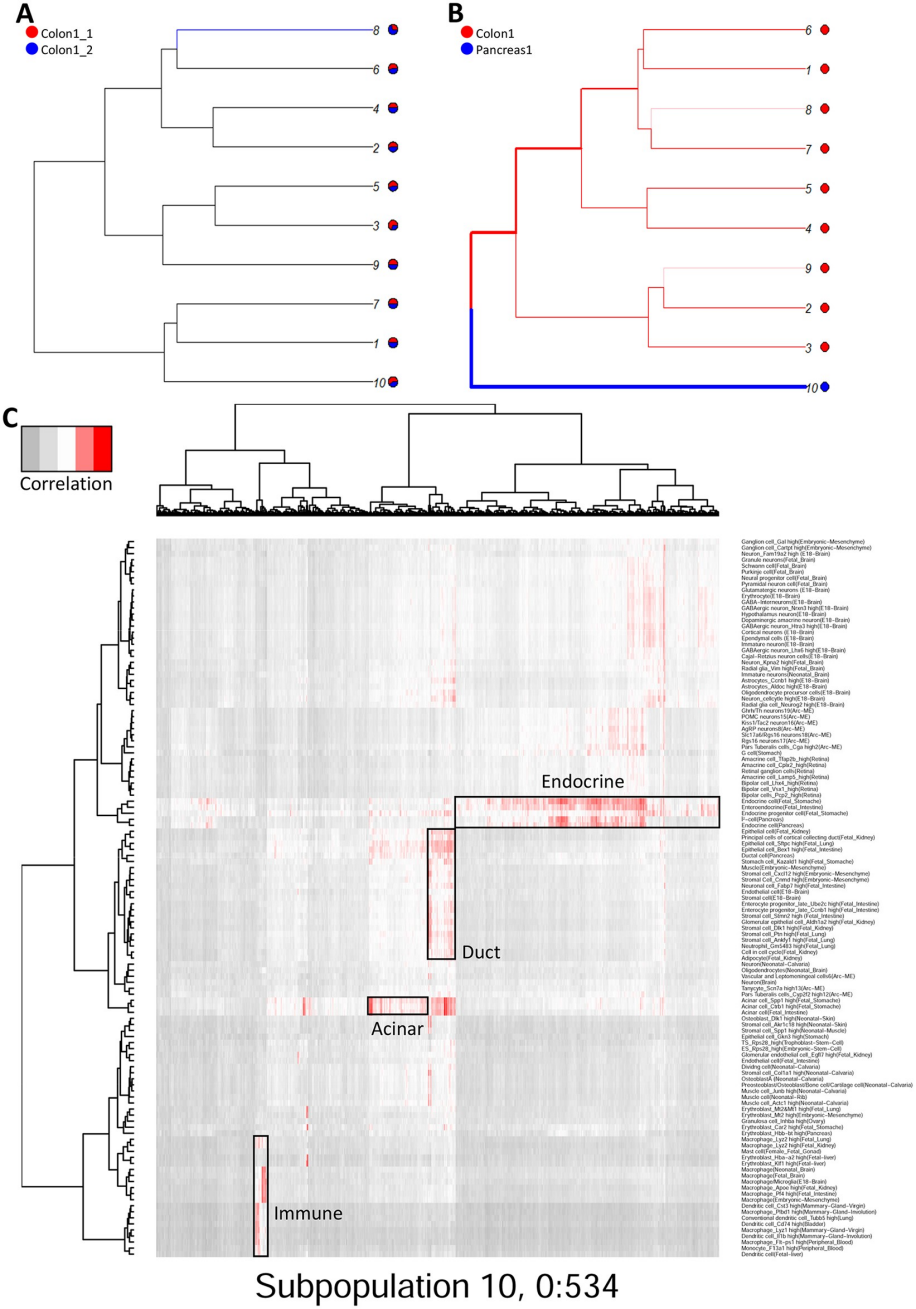
Cells that drive sc-UniFrac can be intuitively identified. (A, B) Branching structure of two single-cell landscapes being scored by sc-UniFrac (k = 10), with black representing statistically shared branches and blue and red representing statistically unshared branches from each of the colored samples. Thickness of branch is proportional to effect size. Comparing between (A) technical replicates and (B) different tissues. (C) Individual cells (columns) from group 10 of panel B being matched to cell types (rows) referenced from the Mouse Cell Atlas. Heat represents the correlation of gene expression between the cell and the reference using all genes. Data from GSE102698, GSE114044, GSE117616.

While sc-UniFrac can statistically measure the population diversity between two single-cell landscapes, it also provides an easy and intuitive way to identify the cells that drive the differences. The distance-driving cells are either expanding or contracting populations or even newly emerging populations across conditions. Instead of relying on nonzero proportional differences between two samples, which would identify every branch and easily be skewed by outliers, sc-UniFrac uses the incorporated permutation significance tests to detect branches whose proportion shifts in cell populations cannot happen by chance alone (Methods). The branches with significant proportional shifts between two samples are distance-driving cells. Illustrating this concept, sc-UniFrac was performed to demonstrate pairwise comparison of scRNA-seq datasets of colonic and pancreatic tissue with k = 10. As expected, technical replicates of the colon with the smallest sc-UniFrac value have mostly shared branches between them, with only one branch with subtle proportional shifts (Fig 5A). In contrast, comparison of the pancreatic and colonic datasets revealed no shared branches, with every unshared branch being highly significant (Fig 5B). Evaluation of unshared branches can easily pinpoint cell groupings that contribute to sc-UniFrac. Here, we focus on group 10, which was composed entirely of cells from the pancreatic sample. Supervised analysis of differential gene expression revealed the unique gene signatures of these cells compared to colonic populations, which can be identified by canonical marker genes (e.g., group 1 represents deep crypt secretory cells; 2 and 3 are colonocytes; 4 and 5 are goblet cells; 6 are intraepithelial lymphocytes) (S8A Fig). Projection of cells from group 10 onto reference cell type gene expression signatures from the Mouse Cell Atlas [3] revealed that individual cells mapped onto pancreatic acinar cells, duct cells, endocrine cells, and immune cells (Fig 5C). These results demonstrate the utility of the branching feature of sc-UniFrac to statistically determine cell populations that drive differences between single-cell landscapes. Notably, none of the methods that we used above for comparison with sc-UniFrac can perform this task.

### Evaluation of physiologically relevant similarities using sc-UniFrac

**Fig 6:**
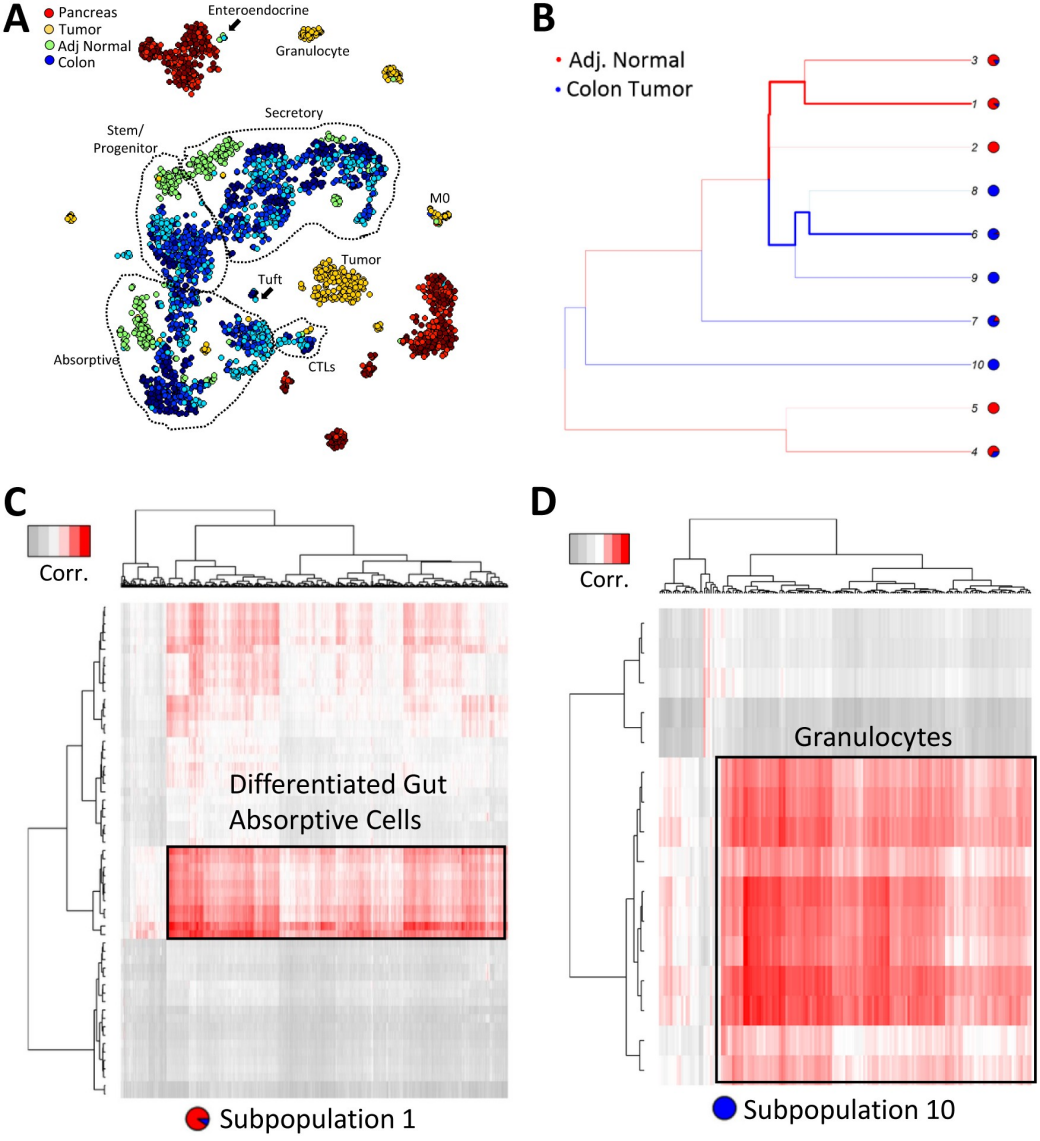
sc-UniFrac identifies unique cellular infiltrates within colonic tumor compared with normal colon. (A) t-SNE plot of multiple replicates of single-cell data from the pancreas, colonic tumor, adjacent normal colon, and normal colon analyzed together. Random sampling of 400 cells from each group. Populations delineated by marker genes. (B) Branching structure of tumor and adjacent normal landscapes scored by sc-UniFrac (k = 10). (C, D) Individual cells (columns) from subpopulations 1 (C) and 10 (D) of panel B being matched to cell types (rows) referenced from the Mouse Cell Atlas. Analysis similar to Fig 5. Data from GSE117615. t-SNE, t-distributed stochastic neighbor embedding.

While sc-UniFrac was able to derive the correct ordering of similarity between normal tissues, we next applied sc-UniFrac to more challenging cases to determine whether it can decipher meaningful dissimilarities in cell diversity arising from the same tissue. Cell type diversity is altered during the process of tumorigenesis as mutations alter signaling pathways to convert cells to abnormal states while, at the same time, additional cell types are recruited to the tumor microenvironment. Nevertheless, cancer cells should harbor some similarities to the cells from the organ of origin while being distinct to cells of other organs. We examined colonic adenoma that are initiated by stochastic loss of the second allele of the tumor suppressor gene Adenomatous Polyposis Coli (*Apc*) in our inducible, stem-cell–driven mouse model (*Lrig1*^*CreERT2/+*^;*Apc*^*fl/+*^) [27,28]. We collected scRNA-seq data using inDrop for both the adenoma and adjacent normal (Fig 6A) and then appended sc-UniFrac analysis of these samples to the existing colon and pancreas analysis. As ranked by sc-UniFrac, the tumor landscape is dissimilar to the normal colon landscape, but it is more similar to the colon than the pancreas landscape, lying somewhere in the middle (Fig 3D). This relationship can be approximated on principal component analysis (PCA) plots (S8B Fig), in which global relationships between data points are better represented. Because all tumor cells would have activated Wnt signaling, there was minimal overlap between the tumor cell and normal cell landscapes, as expected. We performed experiments with control replicate samples to confirm minimal batch effects in this comparison. As such, adjacent normal colon was intermingled among normal colon samples, highlighting its normal phenotype.

The distance-driving cells between tumor and adjacent normal were identified by branches with significant proportional shifts. For simplicity, we selected two subpopulations for further characterization: subpopulation 1, which is the most skewed towards adjacent normal colon, and subpopulation 10, which is composed entirely of cells from the tumor (Fig 6B). Subpopulation 1, when matched to the Mouse Cell Atlas, consists of differentiated absorptive cells of the gut (Fig 6C). This result corroborates known colonic tumor biology that tumors are characterized by stem/progenitor signatures while the normal colon is overrepresented with differentiated cells. Note that the tumor sample also contributes to this subpopulation but only a very minor proportion, as tumors have differentiated cells at a very low level. Subpopulation 10, which is completely tumor derived, represent granulocytes (Fig 6D). Granulocytes—predominantly neutrophils—are absent in the uninflamed normal colon, while tumors present altered, possibly inflamed, microenvironments with substantial infiltrates.

**Fig 7:**
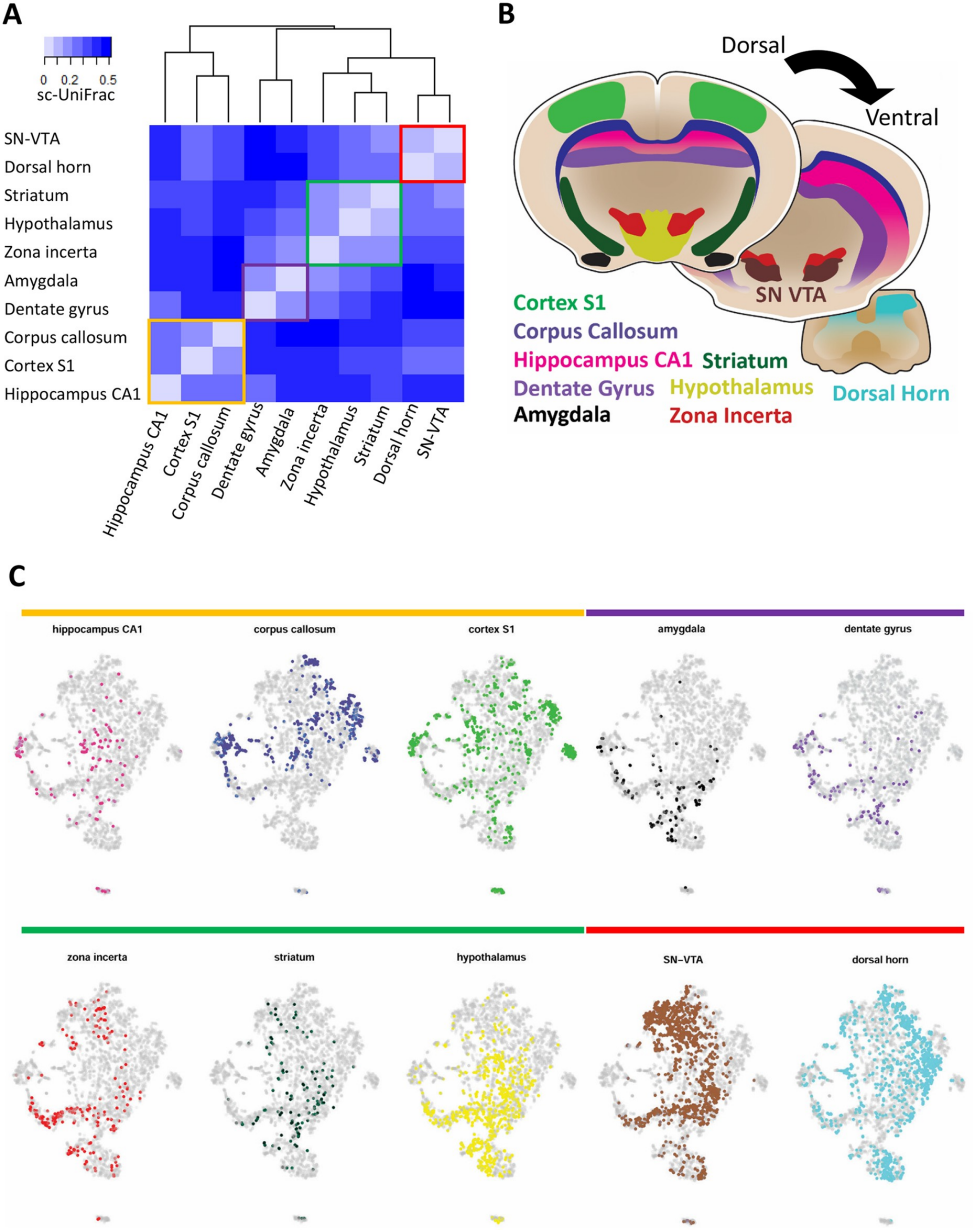
sc-UniFrac groups oligodendrocytes by brain regions. (A) Hierarchical clustering by sc-UniFrac of scRNA-seq data generated from different regions of the brain according to [29]. Heat represents sc-UniFrac distance between two regions. (B) Schematic of brain regions for generating scRNA-seq data. (C) t-SNE plot of data combined from all brain regions, with oligodendrocytes from each region highlighted. Data from GSE75330. scRNA-seq, single-cell RNA sequencing; SN-VTA, substantia nigra and ventral tegmental area; t-SNE, t-distributed stochastic neighbor embedding.

Next, we applied sc-UniFrac to analyze a scRNA-seq dataset describing oligodendrocyte progenitor cells (OPCs) that have been isolated from distinct regions of the mouse brain by microdissection [29]. While region-based information was provided, region-specific differences in OPC subpopulations were not identified in the original manuscript. We analyzed the cells from various brain regions together as in the original manuscript, with the assumption that technical variation between regions has been well-controlled for. Clustering by sc-UniFrac distance, we identified that OPCs grouped together on a dorsal (cortex S1, corpus callosum, hippocampus CA1) to ventral (dentate gyrus, amygdala, zona incerta, striatum, hypothalamus, substantia nigra and ventral tegmental area [SN-VTA], dorsal horn) axis globally (Fig 7A). Looking at more local clustering, we observed grouping of the dentate gyrus and amygdala OPCs, similar to previous work (labeled as “immature” there), while SN-VTA and dorsal horn OPCs grouped together (labeled as “mature” and also physically the most posterior regions of the central nervous system assayed) (Fig 7A). In addition, sc-UniFrac revealed that cortex S1, corpus callosum, and hippocampus CA1 clustered together, while the zona incerta, striatum, and hypothalamus formed another cluster (Fig 7A). These regions develop from pallium-derived and subpallium-derived tissues, respectively (Fig 7B). The groupings by sc-UniFrac can be visually observed in t-SNE plots, supporting our analysis (Fig 7C), although conclusions cannot be definitively drawn by visual inspection alone. Hence, sc-UniFrac was able to provide biologically meaningful results for relating OPCs from different regions of the brain.

### Quantitative evaluation of batch-correction methods by sc-UniFrac

The presence of batch effects is a significant and common problem in scRNA-seq experiments, by introducing systematic error and masking underlying biological signals. Removal of batch effects is generally required prior to downstream analysis. Many methods and tools have been developed for batch correction [30–33]. Some methods have been successfully used in bulk RNA-seq [30,32], while other methods were recently developed and specially designed for scRNA-seq [31,33]. While the suitability of batch-correction methods may depend on the distribution of data that vary from dataset to dataset, the universality of such methods is undefined given that there is no quantitative, objective metric to evaluate batch effect correction in scRNA-seq data. sc-UniFrac, a quantitative measure of cell population diversity in single-cell landscapes, provides a sensitive and objective way to assess the performance of batch-correction methods.

We compared three batch removal methods, limma, ComBat, and MNN, on three scRNA-seq datasets. Limma and ComBat have been widely used for batch correction in bulk experiments, which fit a linear model to determine and then correct the batch effect for each gene [30,32]. MNN first identifies mutual nearest neighbor pairs between batches and then uses these pairs to estimate the batch effect in scRNA-seq data [34]. MNN is expected to perform well when population composition is different across batches. The evaluation of these methods was performed on the following three scRNA-seq datasets: (1) human embryonic kidney 293 (HEK293) cells prepared fresh and cryopreserved from two batches [35], (2) our three technical replicates of mouse colonic epithelium, and (3) two separate studies of mouse gastrulation [36,37].

**Fig 8:**
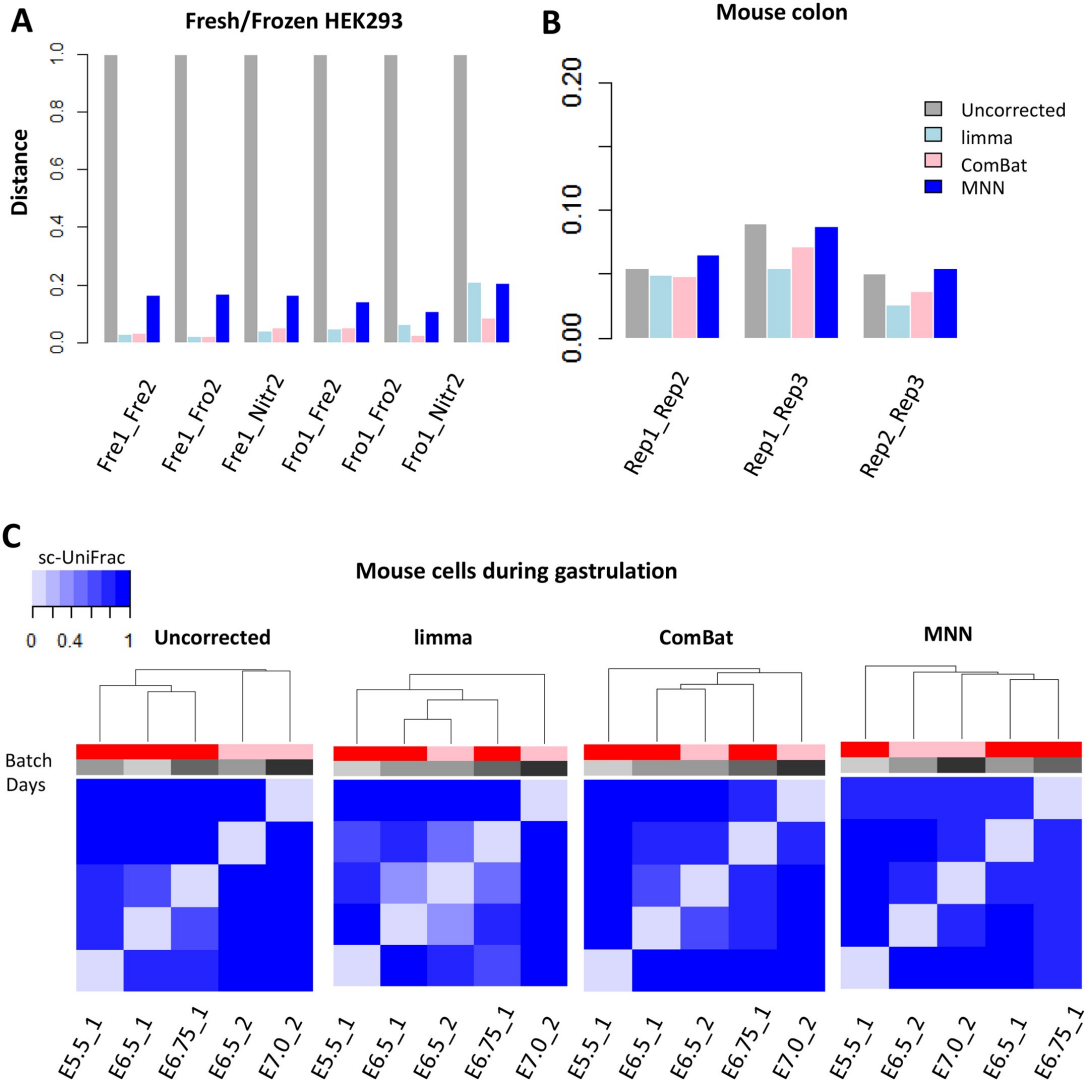
sc-UniFrac can benchmark batch effect removal approaches. (A) sc-UniFrac distance calculated comparing uncorrected and batch-corrected scRNA-seq datasets of HEK293 cells fresh, frozen at −80 °C, or liquid nitrogen flash frozen performed in two different batches (GSE85534) [35]. ComBat, limma, and MNN were used for batch correction. (B) sc-UniFrac distance calculated similar to panel A for technical replicates of the mouse colonic epithelium scRNA-seq data (GSE102698). (C) Hierarchical clustering by sc-UniFrac of uncorrected or batch-corrected scRNA-seq data depicting murine gastrulation from two different studies [36,37]. A gradation of similarity, and hence clustering, was expected over developmental times from the earliest development stage (E5.5) to the latest stage (E7.5). Data from GSE100597; http://gastrulation.stemcells.cam.ac.uk/scialdone2016. E, embryonic day; Fre, fresh; Fro, frozen at −80 °C; HEK293, human embryonic kidney 293; Nitr, liquid nitrogen flash frozen; scRNA-seq, single-cell RNA sequencing.

For HEK293 cell line data, a small sc-UniFrac—reflecting high similarity—was observed between freshly isolated and cryopreserved samples within the same batch (S9 Fig), indicating minimal technical variation during the cryopreservation process consistent with the original findings [35]. In contrast, a large sc-UniFrac was observed between two batches, indicating a strong batch effect similar to the original manuscript (S9 Fig). All three methods, limma, ComBat and MNN, decreased the sc-UniFrac distance, indicative of batch effect correction (Fig 8A and S10A–S10C Fig). Among them, limma and ComBat decreased the distances to those approaching to zero, suggesting that batch effects have been completely removed.

In the technical replicates of the mouse colonic epithelium, only minimal batch effects were observed as indicated by our previous analyses. Due to the initial small differences between batches, limma, ComBat, and MNN only moderately removed batch effects further, as seen in the decrease in sc-UniFrac between replicates 1 and 2, and between replicates 1 and 3 (Fig 8B and S10D–S10F Fig). Batch effects were initially minimal between replicates 2 and 3. In this case, limma and ComBat successfully removed the batch effects (reduced sc-UniFrac to zero), while MNN failed to do so but instead introduced additional systematic bias (sc-UniFrac increased) (Fig 8B).

scRNA-seq data of mouse cells during gastrulation were obtained from two studies [36,37], which used plate-based Smart-seq2 and G&T-seq (genome and transcriptome sequencing), respectively, which introduced large technical variation. The first study generated scRNA-seq data from mouse embryos at E5.5, E6.5, and E6.75, and the second focused on mouse embryos at E6.5 and E7.0. sc-UniFrac generated from the uncorrected data indicated that the two datasets clustered by studies and not by developmental stages, revealing strong technical variation between the two studies (Fig 8C and S11A Fig). After applying limma, ComBat, and MNN, sc-UniFrac indicated that the batch-correction methods removed the technical variation, with cells no longer clustering by studies but by developmental stages. Among the three methods, both limma and ComBat were able to arrange cells chronologically from the earliest development stage (E5.5) to the latest (E7.5), whereas the ordering of samples processed by MNN was incomplete (Fig 8C). This conclusion is supported by t-SNE analysis in which E6.5_1 and E.6.5_2 clustered together after limma and ComBat but remained separated after MNN (S11B–S11D Fig).

From these results, limma and ComBat both outperformed MNN, probably due to identical population composition across batches in all datasets. One of MNN’s assumptions is that batch effects should be much smaller than biological variation, which may not hold true in these datasets. Additionally, the performance of MNN is dependent on the number of nearest neighbors to consider when identifying mutual nearest neighbor pairs. Choosing the correct parameter would probably improve MNN performance, but this would require prior knowledge of what the correct parameter is. While all methods were able to reduce technical variation, sc-UniFrac was able to quantitatively evaluate the initial batch effects and the performance of batch-correction methods.

## Discussion

We developed a new tool, sc-UniFrac, for quantitatively assessing the dissimilarities in cell population structures between two single-cell landscapes that can be generated by various types of single-cell technologies. Compared with existing methods, sc-UniFrac has distinct advantages, including (1) its ability to objectively and quantitatively assess population diversity differences, (2) its precision by taking population structure into account, (3) its statistical rigor based on the available UniFrac framework, (4) its intuitive and statistically robust method to identify disparate cell populations between samples, (5) its flexibility to analyze multiple samples and to add new samples to current analyses, and (6) its ability to handle any dataset with unlimited dimensional representation and distribution [16]. While sc-UniFrac presents a statistical test to estimate the significance of the observed distance, the resulting p-value should be interpreted with caution because it is sensitive to tree topology and randomization methods [38]. We have demonstrated the validity of sc-UniFrac using gold-standard datasets for which the similarities between datasets are known.

Single-cell technologies provide unprecedented resolution to study heterogeneity in disease, especially in cancer. Intratumor heterogeneity is a key determinant of tumor diagnosis, prognosis, and drug response [39,40]. Although a large amount of effort has been devoted to the genomic [41], transcriptomic [42–44], and proteomic [45] subtyping of cancers in hope for better precision application and/or to better understand the disease, current bulk analyses obscure signals coming from distinct cell populations. Unbiased characterization of cellular diversity in tumor tissues and application of this information to define tumor subtypes provides a unique opportunity to better understand cancer. Subtypes based on tumor heterogeneity refine the subtypes defined by bulk “omic” approaches and may provide additional prognostic and diagnostic value for predicting patient survival and drug response. For current single-cell applications, comparing heterogeneity between multiple samples has been performed manually using t-SNE analysis in conjunction with distinguishing markers to qualitatively match cell populations across samples; however, this is done in a low-throughput fashion with few samples [46]. sc-UniFrac enables quantitative evaluation of cellular diversity among potentially large numbers of samples, which can then be rapidly clustered into different subtypes. Thus, sc-UniFrac can facilitate studies on intratumor and intertumor heterogeneity to reveal the importance of diverse cell populations in tumor progression and drug treatment. Furthermore, data structures generated by sc-UniFrac can be applied to software developed for microbiome research, such as QIIME [47], which will provide single-cell researchers access to advanced analytical tools.

Cell populations will expand, shrink, or emerge as a function of disease subtype, disease progression, or after extrinsic drug perturbations. sc-UniFrac can intuitively identify significantly altered cell populations or states driving compositional difference. Moreover, difference-driving cells can be further analyzed to identify gene expression signatures, and their identities and behaviors can be inferred based on transcriptomes of previously referenced cell types. Introduction of new cells into the landscape as a result of perturbation—e.g., the infiltration of CD8 cytotoxic T cells into a tumor—can be deciphered by matching (or blasting) [48] difference-driving cells against the transcriptomes of reference cell types [3], as sc-UniFrac has demonstrated here.

There is currently a proliferation of single-cell data analysis tools, such that many of them utilize different approaches for achieving the same goal. In response, it is necessary for the single-cell biology community to benchmark the performance of these tools with reference datasets. Batch effect correction is a very important procedure for removing technical variation. The sources of variation can arise from runs on different sequencing lanes, different single-cell encapsulation platforms, ischemic times, or different tissue preparations, even if procedures are performed by the same experimenter. Several tools designed to remove batch effects have been developed specifically for scRNA-seq [21,34,49,50]. A quantitative measure of performance is required for effective benchmarking, and sc-UniFrac now provides a metric by which the similarity between single-cell landscapes of a tissue generated from different batches, before and after batch correction, can be evaluated. While evaluation of a tool on any specific datasets can be performed, it should be noted that the performance of any particular tool depends on the assumptions underlying the algorithm as well as the distribution of the dataset. Thus, different tools may perform better on some datasets than others. More importantly, this work calls attention to the requirement for proper experimental design and controls in scRNA-seq experiments. sc-UniFrac reports differences in single-cell landscapes agnostic to technical (batch) versus biological effects. Similar to other experimental platforms such as bulk RNAseq, if proper controls were not performed or an erroneous experimental design was adopted, technical effects can be confounded with biological effects to arrive at erroneous conclusions. Experimental samples should be prepared and run simultaneously with replicate control samples to assess whether the control samples produce similar landscapes, like we have done here with sc-UniFrac on technical and biological replicates. This is especially important for multi-sample experimental designs conducted over different batches. If technical effects were determined to overwhelm biological variation, appropriate measures—such as batch correction—should be performed prior to further downstream analysis. sc-UniFrac can support the evaluation of these effects, such that multiple single-cell landscapes clustering by batch or biological conditions can be readily evaluated in a quantitative and statistically robust manner. Furthermore, cell populations that contribute to batch effects can be identified through the sc-UniFrac pipeline, which helps delineate sources of technical variations, such as an excess of dying cells in one prep versus another. We have demonstrated various applications of this approach, and we envision its broad usage as increasing number of scRNA-seq datasets are generated.

## Methods

### Ethics statement

Animal experiments were performed under protocols approved by the Vanderbilt University Animal Care and Use Committee and in accordance with NIH guidelines. Wild-type mice (C57BL/6) and tumor-bearing mice (*Lrig1*^*CreERT2/+*^;*Apc*^*fl/+*^) were euthanized in an approved fashion prior to dissection and tissue harvesting. Tumor induction was performed following a previous protocol [28].

### The sc-UniFrac framework

sc-Unifrac is freely available as an R package at https://github.com/liuqivandy/scUnifrac. sc-Unifrac includes the following four main steps:

1. Data processing: scRNA-seq data were first normalized by library size per cell (total number of UMIs) and log-transformed.
2. Tree construction: highly variable genes were selected (user defined: default 500). PCA was performed to reduce the dimensionality while preserving the signal of interest, which reduces the noise and makes the data more tractable both from a statistical and computational point of view (user defined: default = 4). A hierarchical tree representing cell population structure was built by clustering via average linkage, and the upper portion of the tree was defined by cutting off the connections at k clusters (user defined: default k = 10).
3. Quantification of cell population diversity: the sc-UniFrac distance was calculated by weighted branch sharing, and statistical significance was assessed by permutation testing.
4. Identifying populations that drive sc-UniFrac by querying the shared branching structure: gene expression signatures were derived for matching against reference cell type signatures.

The sc-Unifrac package provides the following two functions: (1) pairwise comparisons and (2) multi-sample comparisons. In pairwise comparison, sc-Unifrac generates a report to summarize the results, including the sc-UniFrac distance on population diversity, statistical significance, cell population structures, gene expression signatures in each altered population, and their match to reference cell types (S1 Report). In multi-sample comparisons (*n* samples), sc-Unifrac generates an *n*-by-*n* pairwise distance matrix, a corresponding statistical significance matrix, a hierarchical tree, and a table of counts per cluster per sample.

### sc-UniFrac distance calculation

sc-UniFrac (*D*) is calculated as:

$$D=\frac{\sum_{i}^{n}b_{i}\times |\frac{A_{i}}{A_{T}}-\frac{B_{i}}{B_{T}}|}{\sum_{j}^{n}d_{j}\times |\frac{A_{j}}{A_{T}}+\frac{B_{j}}{B_{T}}|}$$

$$pvalue=\frac{\left|D^{*}\geq D\right|}{N}$$

Here, *n* is the total number of branches in the tree. *b*_*i*_ is the length of branch i. *A*_*i*_ and *B*_*i*_ are the number of cells that descend from branch *i* in the two samples A and B, respectively. *A*_*T*_ and *B*_*T*_ are the total number of cells in two samples A and B, respectively. $\sum_{j}^{n}d_{j}*|\frac{A_{j}}{A_{T}}+\frac{B_{j}}{B_{T}}|$ is the average distance of each cell from the root, used to normalized the distance from 0 to 1. D* is the distance based on permuted data, while *D* is the observed distance. *N* is the total number of permutations. A distribution of distance is obtained with a *p*-value that reflects the probability that the permuted distances are greater than or equal to the observed distance by chance.

Unshared branches are occupied by populations with statistically significant shifts between samples. The proportion shift of a cell population *i* is defined as ${ps}_{i}=|\frac{A_{i}}{A_{T}}-\frac{B_{i}}{B_{T}}|$, with statistical significance achieved if *ps*_*i*_ > ps*, where ps* is the proportion shift in permuted datasets.

### Signature matching to reference cell types

The signature composed of under- or overexpressed genes associated with a cell population was defined by limma comparisons with other populations. For predicting cell types, each cell of a given cluster was matched to the 894 cell type references from the Mouse Cell Atlas [3]. Matching was performed by deriving a Pearson correlation of all genes between the query cell and the reference cell type transcriptome.

### Modified scoring of p-Creode trajectories

The p-Creode score was originally designed to compare p-Creode trajectories as a means to evaluate robustness of the calculated trajectories and also to arrive at a representative trajectory over multiple bootstrapped runs on the same single-cell dataset. Thus, it was developed to assess the dissimilarity between trajectories of largely the same sizes. Here, we use p-Creode scoring to compare different datasets, which can generate trajectories of different sizes. To make the p-Creode score size-invariant, we modified how nodes are transformed from one graph to another while maintaining the scoring approach outlined in [19]. Previously, when a node was not contained in both graphs, a node transformation was performed by translating the node in the second graph to the closest node in the first graph with a penalty (S6A Fig). To eliminate excess penalty when a dense graph is transformed into a sparse graph and vice versa (S6C Fig), the scoring routine was updated to allow for transformations into the edges as well as nodes in the reference first graph. More formally, the transformation penalty is the minimum distance to the closest edge projection between two nodes in opposing graphs plus the remaining graph edge distance to the closest node along the path of the pairwise comparison (S6B Fig). The code and data for reproducing the analysis is at https://github.com/KenLauLab/pCreode_Comparison_Across_Datasets.

### Batch-correction methods

We compared three batch-correction methods, limma, ComBat, and MNN. For limma, we used the removeBatchEffect function in the limma package, which fits a linear model to the data and then removes the component due to the batch effects [32]. For ComBat, we used the ComBat function in the sva package, which uses Empirical Bayes methods to adjust for both the mean and variance differences across the batches [30]. MNN identifies the mutual nearest neighbors between batches and uses them to estimate and remove the batch effect [34]. We performed the mnnCorrect function in the scran package. We set the number of nearest neighbors to consider to be 20.

### scRNA-seq data sources

#### T-cell differentiation CyTOF dataset [25]

This is a mass cytometry dataset characterizing the mouse thymus during T-cell development, such that lymphoid progenitors differentiate to either CD8^+^ or CD4^+^ cells. Data on about 250,000 cells on 37 surface markers and transcription factors were generated. We removed the DN cell population and included only CD8^+^ or CD4^+^ cells labeled by Wishbone. We then simulated population mixtures by randomly sampling CD8^+^ and CD4^+^ cells.

#### Myeloid differentiation scRNA-seq dataset [26]

This is a sc-RNAseq data conducted using MAR-seq on myeloid cell differentiation. A total of 4,423 cells were included after filtering. Erythrocytes and myeloid progenitor cells were further identified and gated using canonical markers. We then simulated population mixtures by randomly sampling erythrocytes and myeloid progenitor cells. Raw data can be assessed in GEO with accession number GSE72857.

#### Fresh and frozen HEK293 scRNA-seq datasets [35]

Single-cell transcriptomes of the fresh and cryopreserved HEK293 cells were generated by MARS-seq, which included about 50 cells in each sample. The UMI-filtered read counts were downloaded from GEO with accession number GSE85534.

#### Technical and biological replicate scRNA-seq datasets from mouse colon and pancreas tissues newly generated

scRNA-seq data of colonic, colonic tumor, and pancreatic tissues were generated by inDrop platform, with datasets sizes ranging from approximately 500 to 1,800 cells (Methods described below). The UMI-filtered read counts and raw data are available from GEO with accession numbers GSE102698, GSE114044, GSE117615, and GSE117616.

#### Mouse gastrulation scRNA-seq datasets [36,37]

scRNA-seq data of mouse cells during gastrulation were obtained from two studies. Mohammed and colleagues isolated single cells from mouse embryos at different stages and generated scRNA-seq data using G&T-seq. We selected data from the E5.5 (267 cells), E6.5 (168 cells), and E6.75 stages (82 cells). Count tables were downloaded from GEO with accession number GSE100597. Scialdone and colleagues used Smart-seq2 to profile 1,205 cells of gastrulating mouse embryos. We selected data from the E6.5 (502 cells) and E7.0 stages (138 cells). Gene counts were downloaded from http://gastrulation.stemcells.cam.ac.uk/scialdone2016. Ensembl gene IDs were mapped to mouse gene symbols using the biomaRt package.

#### Oligodendrocyte scRNA-seq datasets from mouse brain regions [29]

scRNA-seq data tables from brain regions microdissected from mouse brain sections were downloaded from GSE75330. In total, 5,053 cells were analyzed without filtering, distributed as cortex S1 (613), hippocampus CA1 (112), corpus callosum (591), dentate gyrus (114), dorsal horn (1,127), zona incerta (225), amygdala (135), hypothalamus (754), SN-VTA (1,247), and striatum (135).

### scRNA-seq of colonic and pancreatic tissues

Single-cell suspensions of colonic epithelium were prepared by chelating (3 mM EDTA; 1 mM DTT) distal colon segments at 4°C for 45 minutes followed by shaking off crypts [19,51]. Isolated crypts were then dissociated into single cells using a DNase1/collagenase enzymatic cocktail (2.5 mg/mL DNase1, 2 mg/mL collagenase) at 37°C for 20 minutes. Crypt fragments were further mechanically dissociated into single cells using a 27.5-gauge needle. Cell suspensions were washed 2 times with cold PBS to remove debris and were enriched for live cells using a Miltenyi MACS dead cell removal kit. Live cell concentration was counted based on Trypan Blue positive cells, and a solution of 150,000 cells/mL was prepared for encapsulation. To maintain live cell viability, 18 ul of Optiprep was added per 100 ul of cell solution prior to encapsulation.

Dissociation of pancreatic buds from E14.5 control embryos was performed using previously published protocols [52]. Briefly, pancreatic buds were dissected from control embryos and trypsinized followed by flow sorting. Single-cell suspensions from multiple embryonic buds were prepared, and a cell solution of 20,000 cells was prepared for encapsulation.

Single-cell encapsulation was performed using the inDrop platform (1CellBio) with an in vitro transcription library preparation protocol, as previously described [1]. inDrop utilizes CEL-Seq in preparation for sequencing and is summarized as follows: (1) reverse transcription (RT), (2) ExoI nuclease digestion, (3) SPRI purification (SPRIP), (4) single strand synthesis, (5) SPRIP, (6) T7 in vitro transcription linear amplification, (7) SPRIP, (8) RNA fragmentation, (9) SPRIP, (10) primer ligation, (11) RT, and (12) library enrichment PCR. Number of cells encapsulated was calculated by approximating the density of single-cell suspension multiplied by bead loading efficiency during the duration of encapsulation. Each sample was estimated to contain approximately 2,500 encapsulated cells.

Following library preparation, as described above, the samples were sequenced using Nextseq 500 (Illumina) using a 150 bp paired-end sequencing kit in a customized sequencing run [19]. After sequencing, reads were filtered, sorted by their designated barcode, and aligned to the reference transcriptome using InDrops pipeline. Mapped reads were quantified into UMI-filtered counts per gene, and barcodes that correspond to cells were retrieved based on previously established methods [1]. From approximately 2,500 cells encapsulated, approximately 1,800 cells were retrieved per sample.

### Dissociation of colonic tumors

Dissociation of colonic adenomas was performed in a two-phase process. In the first stage, colon adenomas were dissected from the distal colon and washed in ice-cold PBS. The tumors were digested in DMEM containing 2 mg/mL collagenase type II at 37 °C for 1 hour or until fragments had dispersed. The tumor tissue suspension was washed in ice-cold PBS and filtered through a 40 μm filter. Tumor epithelial crypts retained by the filter were collected and resuspended in PBS while the flow-through was discarded. The tumor epithelial fraction was filtered again through a 100 μm filter to remove undigested fragments, and the flow-through was collected. In the second stage, isolated tumor epithelial crypts were further digested into single cells for encapsulation similar to above.

### Experimental design

Technical replicates were different single-cell encapsulations collected from the same mouse colon but prepared and sequenced on different days. Biological replicates were tissues collected from different mice on different days but sequenced in the same run.

### Computational performance test of sc-UniFrac

sc-UniFrac was applied to two scRNA-seq datasets to test the time and memory cost, each composed of 25,507 genes and 1,000 cells. All tests were run without parallel computation on a Windows (Microsoft; https://www.microsoft.com/en-us/) desktop with an Intel(R) Xeon(R) CPU E5-2620 0 at 2 GHz and 32 GB memory.

With default parameters (500 highly variable genes, 4 PCs, and 10 clusters), sc-UniFrac took about 25 seconds to calculate the distance and statistical significance. The maximum memory used by sc-UniFrac was about 800 MB. The time and memory used only increased nominally with increasing numbers of clusters, increasing numbers of PCs, and increasing numbers of genes used. As expected, the running time increased linearly with the number of cells in each dataset (Table 1).

**Table 1:** Computational time of sc-UniFrac on two datasets composed of 25,507 genes.

Number of cells in each dataset	Running time
1,000	25 s
2,000	49 s
3,000	76 s
4,000	99 s

## Supporting information

S1 Fig t-SNE plot of two 1,000-cell populations simulated by resampling from CD4 and CD8 cells. One cell population (N1) included only CD8 cells, while the other cell population N2 was composed of proportional mixtures of CD4 and CD8 cells (S1 Data) [25]. (A) N2 = 100% CD4 cells. (B) N2 = 100% CD8 cells. (C) N2 = 95% CD8 cells; 5% CD4 cells. (D) N2 = 50% CD8 cells; 50% CD4 cells.
(TIF)

S2 Fig Distribution of data when dataset size is imbalanced. t-SNE plots of similar simulations as in S1 Fig, with N2 being 100% CD8 cells, 50/50 CD8/CD4 cells, and 100% CD4 cells, going from left to right (S1 Data). Altering the size of N2 to be (A) 500 and (B) 100. N1 remains at 1,000 cells.
(TIF)

S3 Fig Sensitivity and robustness analysis of sc-UniFrac using simulated sc-RNAseq data. (A) Two groups (N1 and N2) of 500 cells were selected from erythrocyte and myeloid progenitor cells identified in the Paul and colleagues dataset (S2 and S3 Data) [26]. N1 is always composed of 100% erythrocytes, while N2 is composed of erythrocytes and different proportions of myeloid progenitor cells (indicated on x-axis); y-axis is the sc-UniFrac distance calculated over *n* = 50 runs with k = 10. Boxes represent the first and third quartiles, and bars represent maximum and minimum values. (B) Sensitivity of sc-UniFrac evaluated by the fraction of incidences that a statistically significant sc-UniFrac distance was returned over *n* = 50 runs, as a function of increasing dissimilarity between N1 and N2 using the same simulation scheme as panel A. (C) Mean sc-UniFrac plotted as in panel A with varying k parameter. (D) Fraction significant sc-UniFrac detected plotted as in panel B with varying k parameter. (E) Mean sc-UniFrac plotted as in A with N1 = 500 but a varying N2 size to determine the effect of dataset size imbalance on sc-UniFrac. (F) Fraction significant sc-UniFrac detected plotted as in B with N1 = 500 and varying N2 size.
(TIF)

S4 Fig Bulk analysis to demonstrate the ordering of similarity between scRNA-seq data from technical and biological replicates of the colon versus the pancreatic islet. Gene correlation analysis in which scRNA-seq data were averaged to generate bulk values. Each data point (on the lower triangle plots) represents a gene whose log expression level was plotted between the two samples being compared. Upper triangle plots are calculated correlation coefficients.
(TIF)

S5 Fig Analysis of scRNA-seq data from technical and biological replicates of the colon, and the pancreatic islet. *Krt20* depicting the absorptive lineage, *Muc2* depicting the secretory lineage, and *Cd8a* depicting immune cells overlaid on t-SNE plots of scRNA-seq data generated from the adult murine colonic mucosa with (A) technical and (B) biological replicates. (C) Hierarchical clustering by sc-UniFrac of scRNA-seq landscapes of the E14.5 pancreatic islet and adult colonic mucosa (indicated by tissue label), with technical and biological replicates (indicated by mouse label). Heat represents sc-UniFrac distance between two samples.
(TIF)

S6 Fig Differences between trajectories constructed from continuous single-cell data revealed by p-Creode scoring. (A) Scheme of old node-to-node projection strategy used for the previous p-Creode scoring approach [19]. Dotted line represents Euclidean distance penalty of each transformation. Green and red nodes are from different trajectories. (B) Scheme of new node-to-edge projection strategy used for the current p-Creode scoring approach. (C) Demonstration of excess penalization using the previous p-Creode scoring strategy when there is an imbalance in dataset size resulting in different numbers of nodes in the trajectory (top) versus more realistic penalization with the current approach (bottom). (D) Hierarchical clustering by p-Creode scoring of trajectories generated from scRNA-seq data of E14.5 pancreatic islet (green—biological replicates) and adult colonic mucosa (red—technical and biological replicates). *N* = 100 resampled p-Creode runs for each dataset were performed and then analyzed together in a single clustering analysis. Heat represents the p-Creode score between two trajectories.
(TIF)

S7 Fig p-Creode trajectory analysis of scRNA-seq data from technical and biological replicates of the colon, and the pancreatic islet. (A) *Krt20* depicting colonocytes, *Reg4* depicting deep crypt secretory cells, and *Myc* depicting stem and progenitor cells overlaid on a representative p-Creode trajectory of scRNA-seq data generated from the murine colonic epithelium. (B) Representative p-Creode trajectories depicting colonic and pancreatic islet differentiation. Outlined lineages were identified with canonical markers. Overlay of *Muc2* transcript level, which was not expressed in the pancreatic islet.
(TIF)

S8 Fig Gene signature extraction and single-cell landscape ordering using sc-UniFrac. (A) Differential expressed gene identified by limma for each of the 10 groups in Fig 5. (B) PCA plot of multiple replicates of single-cell data from the pancreas, colonic tumor, adjacent normal colon, and normal colon analyzed together as in Fig 6A.
(TIF)

S9 Fig Comparing scRNA-seq data from frozen or freshly prepared samples from different batches. Hierarchical clustering by sc-UniFrac of scRNA-seq data from cell lines that are prepared differently (GSE85534) [35]. Heat depicts the sc-UniFrac distance between 2 samples. The results are consistent with the original study, which shows that the freezing process did not alter transcriptional profiles. In contrast, batch effects have a larger impact on the transcription profiles than the freezing process.
(TIF)

S10 Fig The effects of batch correction. t-SNE analysis of scRNA-seq data from cell lines prepared from two batches (Frozen 1 and 2) [35] (A) uncorrected, (B) corrected by ComBat, and (C) corrected by MNN. t-SNE analysis of scRNA-seq data from the colonic mucosa from two technical replicates (Replicates 1 and 2). sc-UniFrac distance between the samples and *p*-value noted.
(TIF)

S11 Fig Batch correction applied on data from different studies to align samples according to developmental time. t-SNE analysis of scRNA-seq data depicting mouse gastrulation, with colors representing developmental time and shapes of data points representing the two studies [36,37]. For instance, all red data points should cluster together. Analysis performed on (A) uncorrected, (B) ComBat-corrected, (C) limma-corrected, and (D) MNN-corrected data.
(TIF)

S1 Data Processed data supporting Fig 2. (XLSX)

S2 Data Processed data (erythrocyte) supporting S3 Fig. (CSV)

S3 Data Processed data (myeloid progenitor) supporting S3 Fig. (CSV)

S4 Data Processed data supporting Fig 4. (XLSX)

S1 Report Example of sc-UniFrac-generated report. (PDF)

## Abbreviations

Apc: Adenomatous Polyposis Coli
CyTOF: cytometry time-of-flight
E: embryonic day
EMD: Earth Mover’s Distance
HEK293: human embryonic kidney 293
OPC: oligodendrocyte progenitor cell
PCA: principal component analysis
RT: reverse transcription
scRNA-seq: single-cell RNA-sequencing
SN-VTA: substantia nigra and ventral tegmental area
SPRIP: SPRI purification
t-SNE: t-distributed stochastic neighbor embedding
