# Supplementary figures and images for: Quantitative assessment of cell population diversity in single-cell landscapes
Source: PLoS Biol. 2018 Oct 22;16(10):e2006687. doi: 10.1371/journal.pbio.2006687 (PMC6211764; doi:10.1371/journal.pbio.2006687)

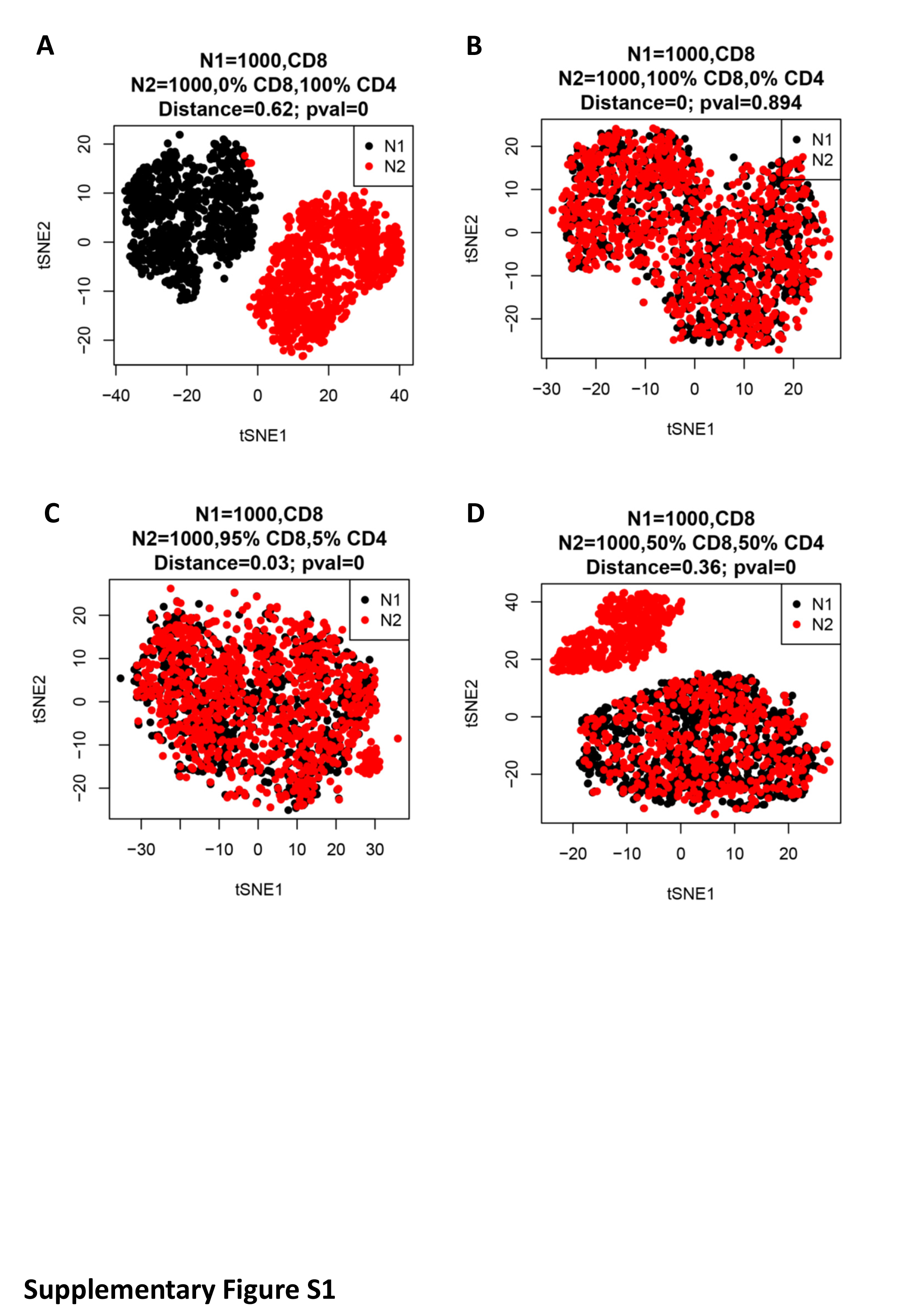

Supplement: S1 Fig — One cell population (N1) included only CD8 cells, while the other cell population N2 was composed of proportional mixtures of CD4 and CD8 cells (S1 Data) [25]. (A) N2 = 100% CD4 cells. (B) N2 = 100% CD8 cells. (C) N2 = 95% CD8 cells; 5% CD4 cells. (D) N2 = 50% CD8 cells; 50% CD4 cells. (TIF) [file pbio.2006687.s001.tif]

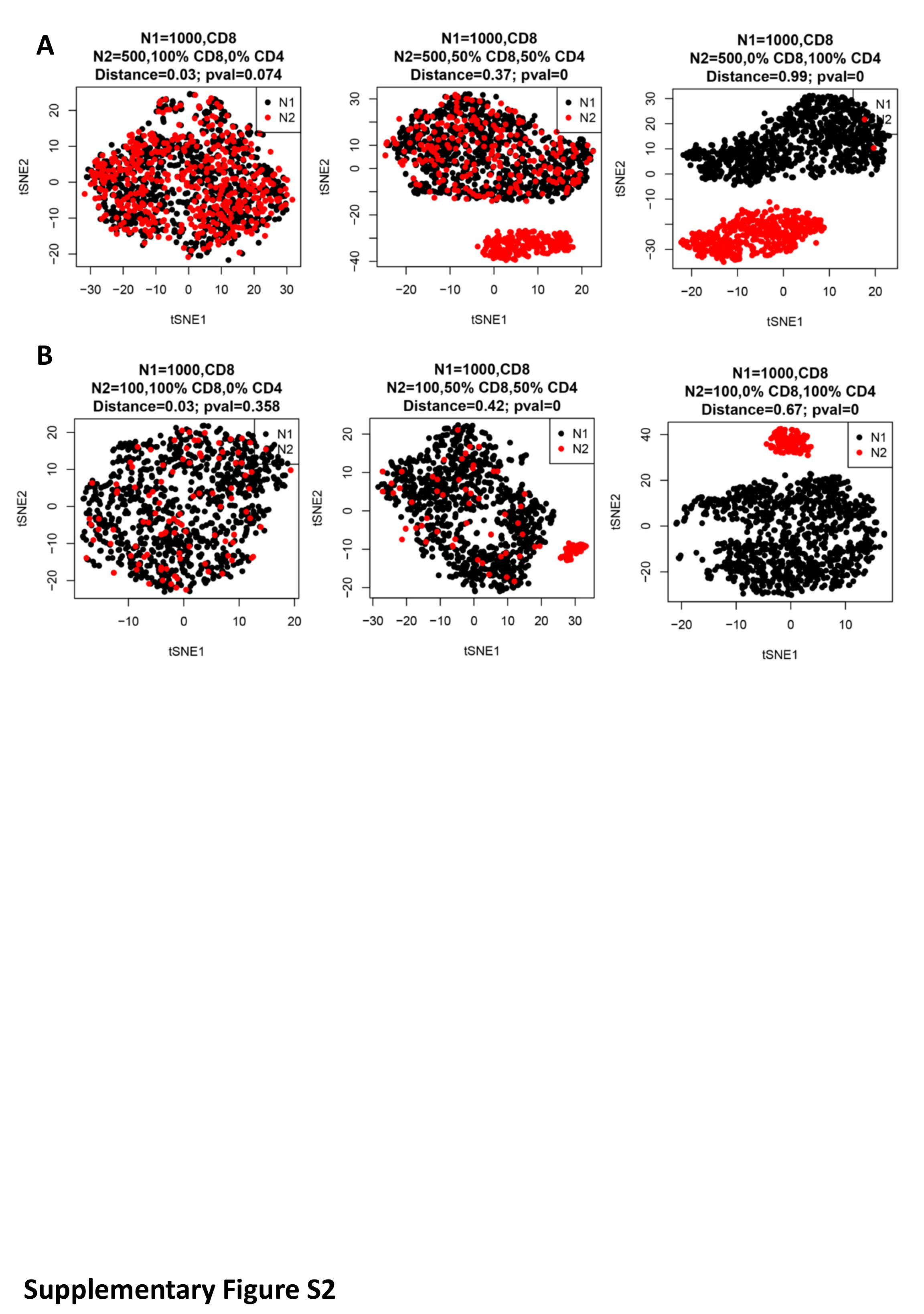

Supplement: S2 Fig — t-SNE plots of similar simulations as in S1 Fig, with N2 being 100% CD8 cells, 50/50 CD8/CD4 cells, and 100% CD4 cells, going from left to right (S1 Data). Altering the size of N2 to be (A) 500 and (B) 100. N1 remains at 1,000 cells. (TIF) [file pbio.2006687.s002.tif]

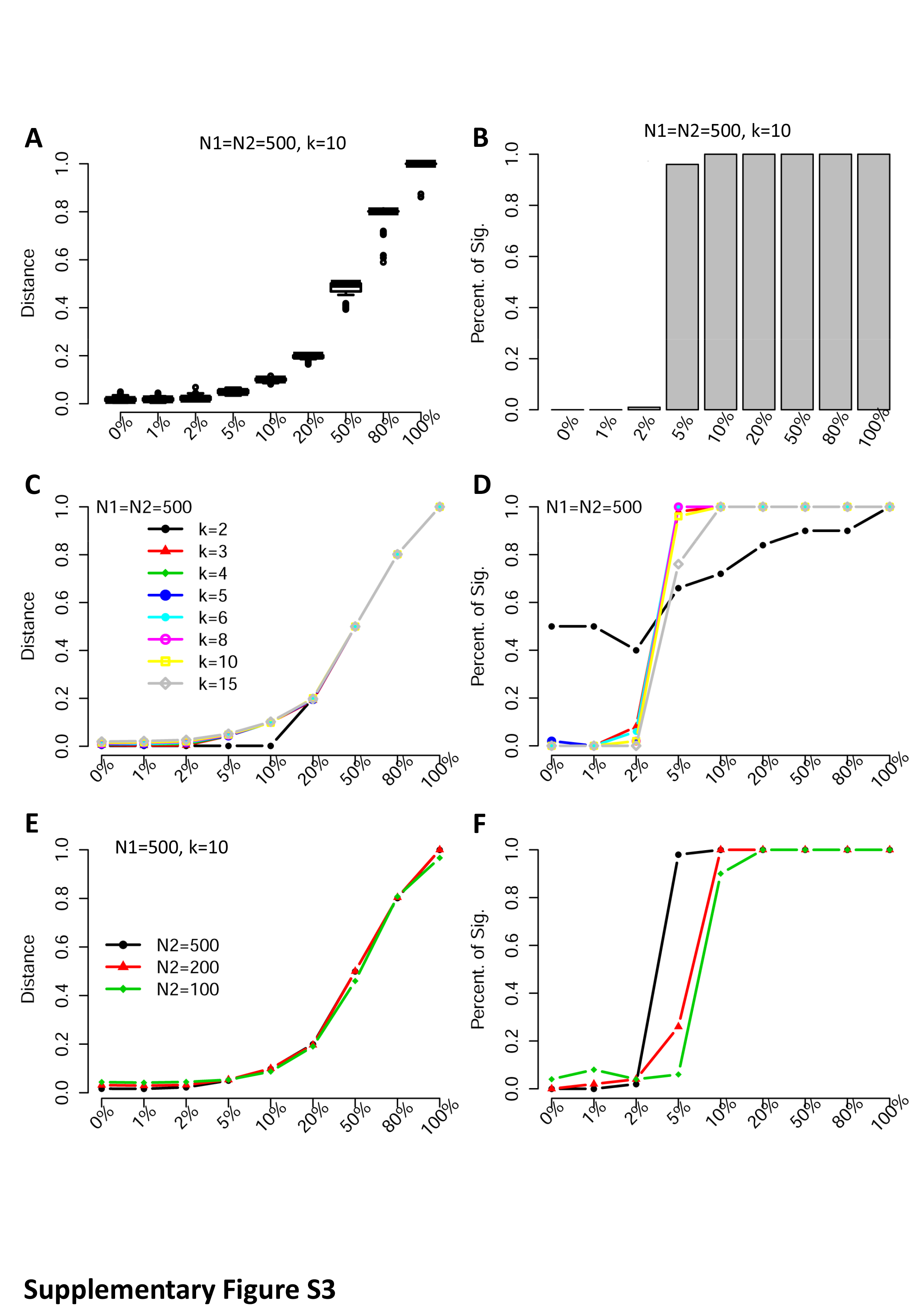

Supplement: S3 Fig — (A) Two groups (N1 and N2) of 500 cells were selected from erythrocyte and myeloid progenitor cells identified in the Paul and colleagues dataset (S2 and S3 Data) [26]. N1 is always composed of 100% erythrocytes, while N2 is composed of erythrocytes and different proportions of myeloid progenitor cells (indicated on x-axis); y-axis is the sc-UniFrac distance calculated over n = 50 runs with k = 10. Boxes represent the first and third quartiles, and bars represent maximum and minimum values. (B) Sensitivity of sc-UniFrac evaluated by the fraction of incidences that a statistically significant sc-UniFrac distance was returned over n = 50 runs, as a function of increasing dissimilarity between N1 and N2 using the same simulation scheme as panel A. (C) Mean sc-UniFrac plotted as in panel A with varying k parameter. (D) Fraction significant sc-UniFrac detected plotted as in panel B with varying k parameter. (E) Mean sc-UniFrac plotted as in A with N1 = 500 but a varying N2 size to determine the effect of dataset size imbalance on sc-UniFrac. (F) Fraction significant sc-UniFrac detected plotted as in B with N1 = 500 and varying N2 size. (TIF) [file pbio.2006687.s003.tif]

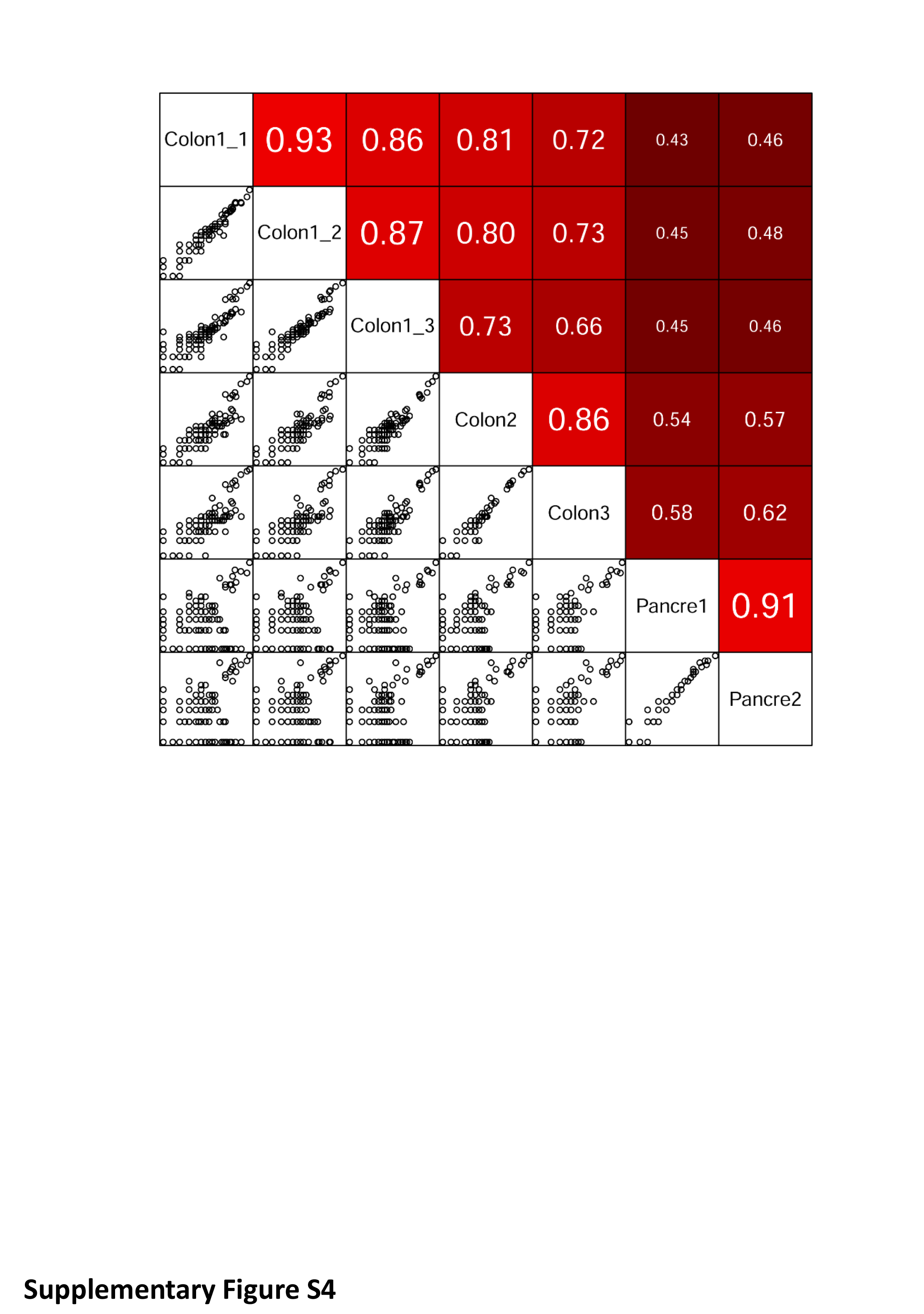

Supplement: S4 Fig — Gene correlation analysis in which scRNA-seq data were averaged to generate bulk values. Each data point (on the lower triangle plots) represents a gene whose log expression level was plotted between the two samples being compared. Upper triangle plots are calculated correlation coefficients. (TIF) [file pbio.2006687.s004.tif]

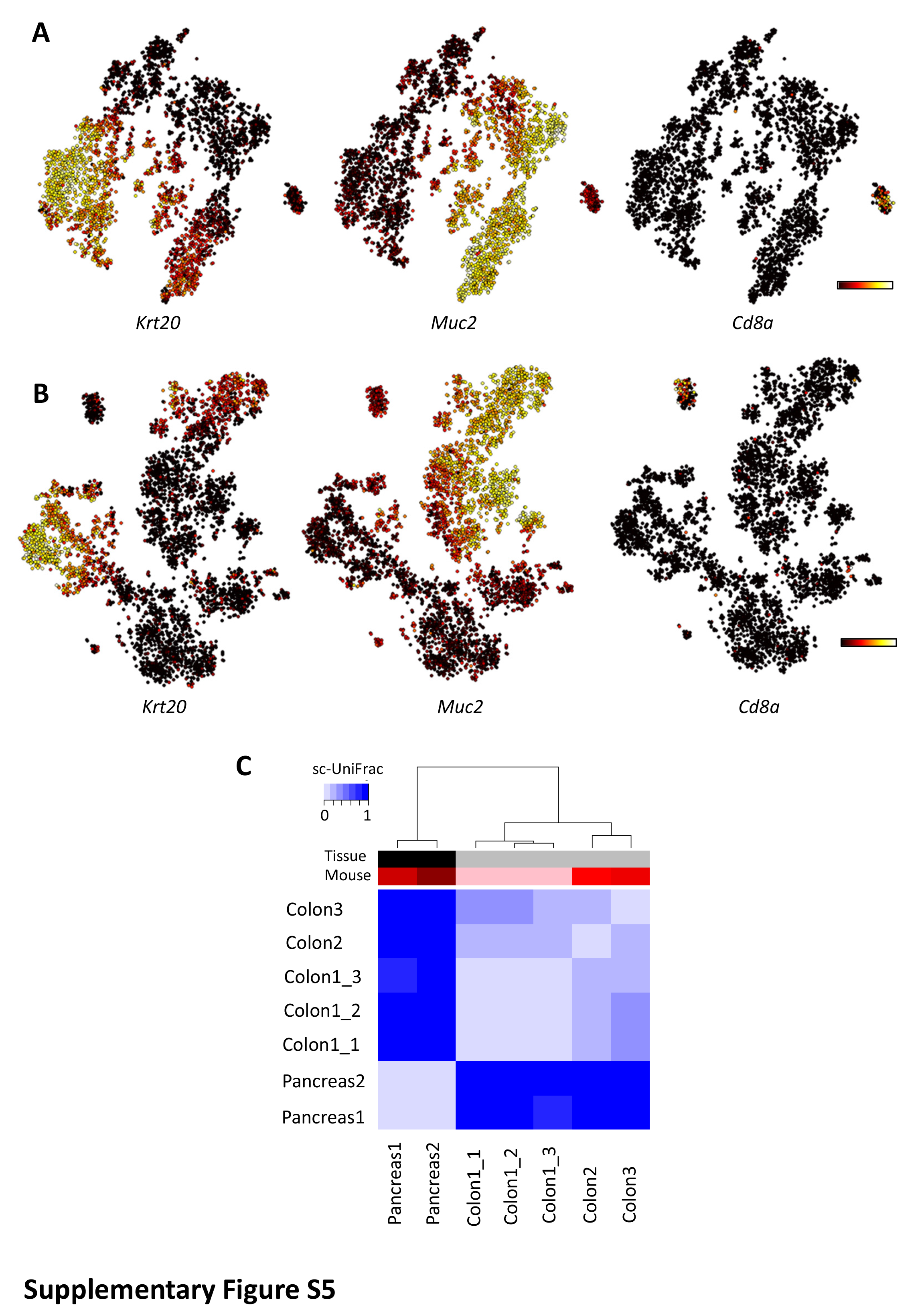

Supplement: S5 Fig — Krt20 depicting the absorptive lineage, Muc2 depicting the secretory lineage, and Cd8a depicting immune cells overlaid on t-SNE plots of scRNA-seq data generated from the adult murine colonic mucosa with (A) technical and (B) biological replicates. (C) Hierarchical clustering by sc-UniFrac of scRNA-seq landscapes of the E14.5 pancreatic islet and adult colonic mucosa (indicated by tissue label), with technical and biological replicates (indicated by mouse label). Heat represents sc-UniFrac distance between two samples. (TIF) [file pbio.2006687.s005.tif]

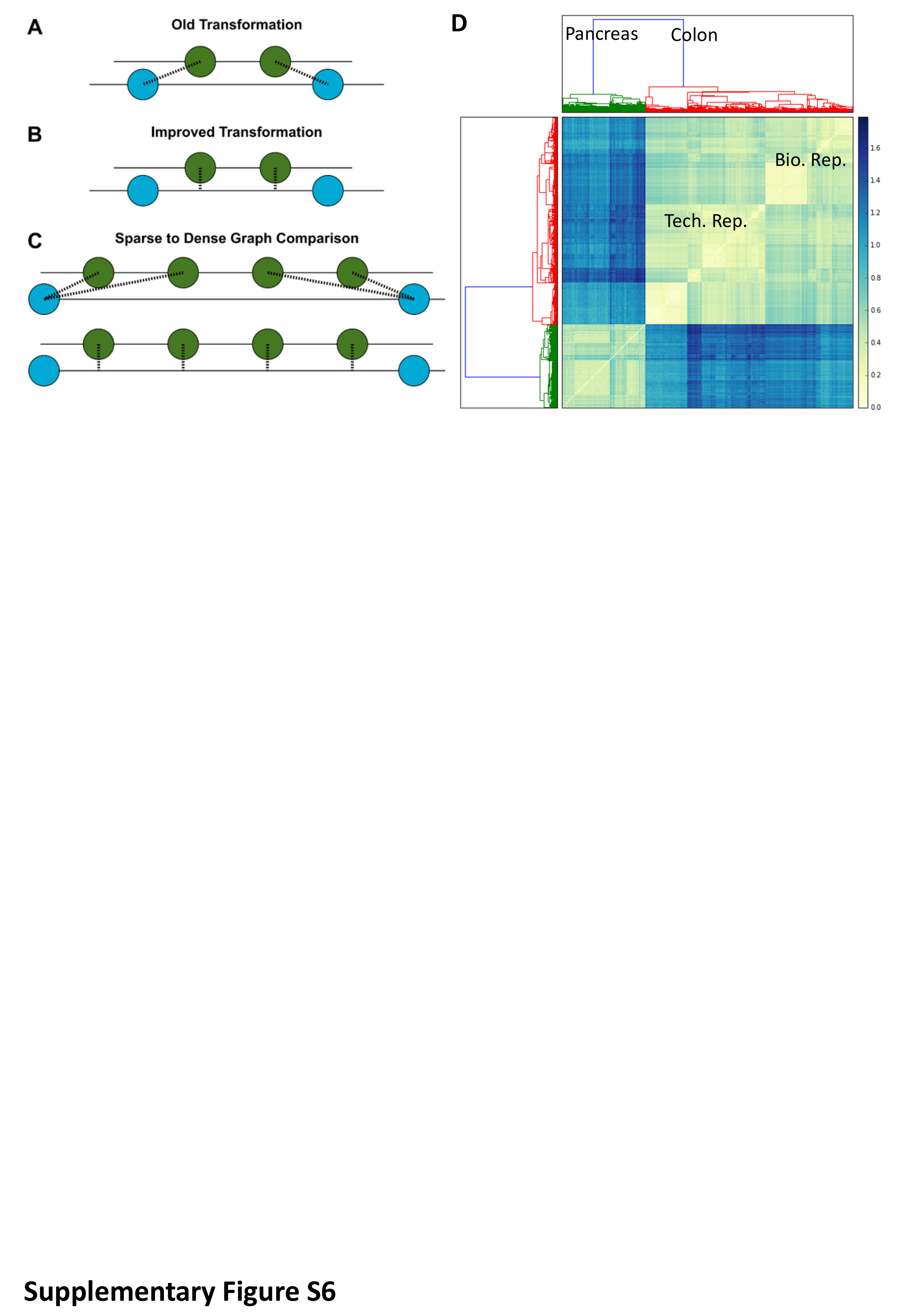

Supplement: S6 Fig — (A) Scheme of old node-to-node projection strategy used for the previous p-Creode scoring approach [19]. Dotted line represents Euclidean distance penalty of each transformation. Green and red nodes are from different trajectories. (B) Scheme of new node-to-edge projection strategy used for the current p-Creode scoring approach. (C) Demonstration of excess penalization using the previous p-Creode scoring strategy when there is an imbalance in dataset size resulting in different numbers of nodes in the trajectory (top) versus more realistic penalization with the current approach (bottom). (D) Hierarchical clustering by p-Creode scoring of trajectories generated from scRNA-seq data of E14.5 pancreatic islet (green—biological replicates) and adult colonic mucosa (red—technical and biological replicates). N = 100 resampled p-Creode runs for each dataset were performed and then analyzed together in a single clustering analysis. Heat represents the p-Creode score between two trajectories. (TIF) [file pbio.2006687.s006.tif]

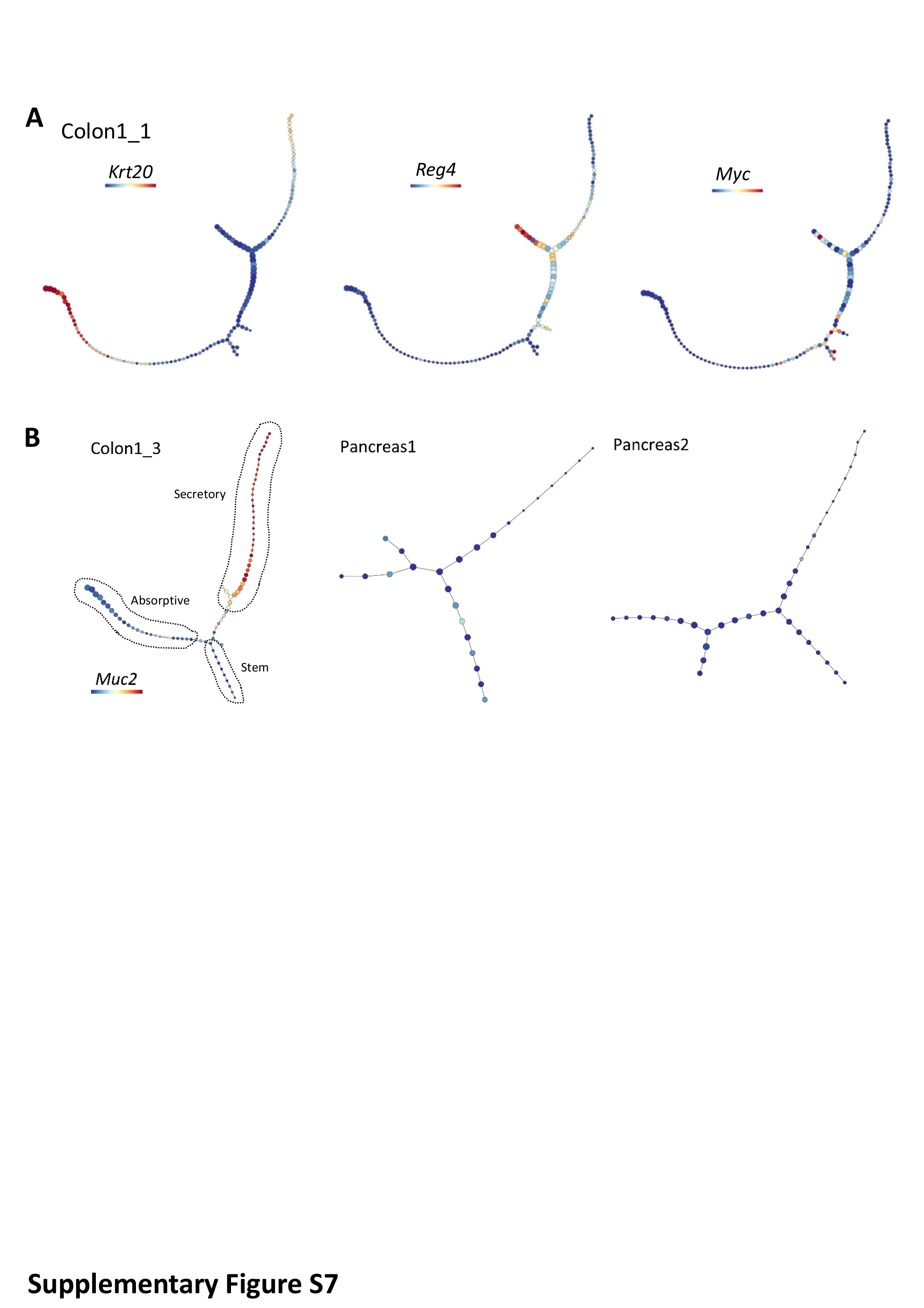

Supplement: S7 Fig — (A) Krt20 depicting colonocytes, Reg4 depicting deep crypt secretory cells, and Myc depicting stem and progenitor cells overlaid on a representative p-Creode trajectory of scRNA-seq data generated from the murine colonic epithelium. (B) Representative p-Creode trajectories depicting colonic and pancreatic islet differentiation. Outlined lineages were identified with canonical markers. Overlay of Muc2 transcript level, which was not expressed in the pancreatic islet. (TIF) [file pbio.2006687.s007.tif]

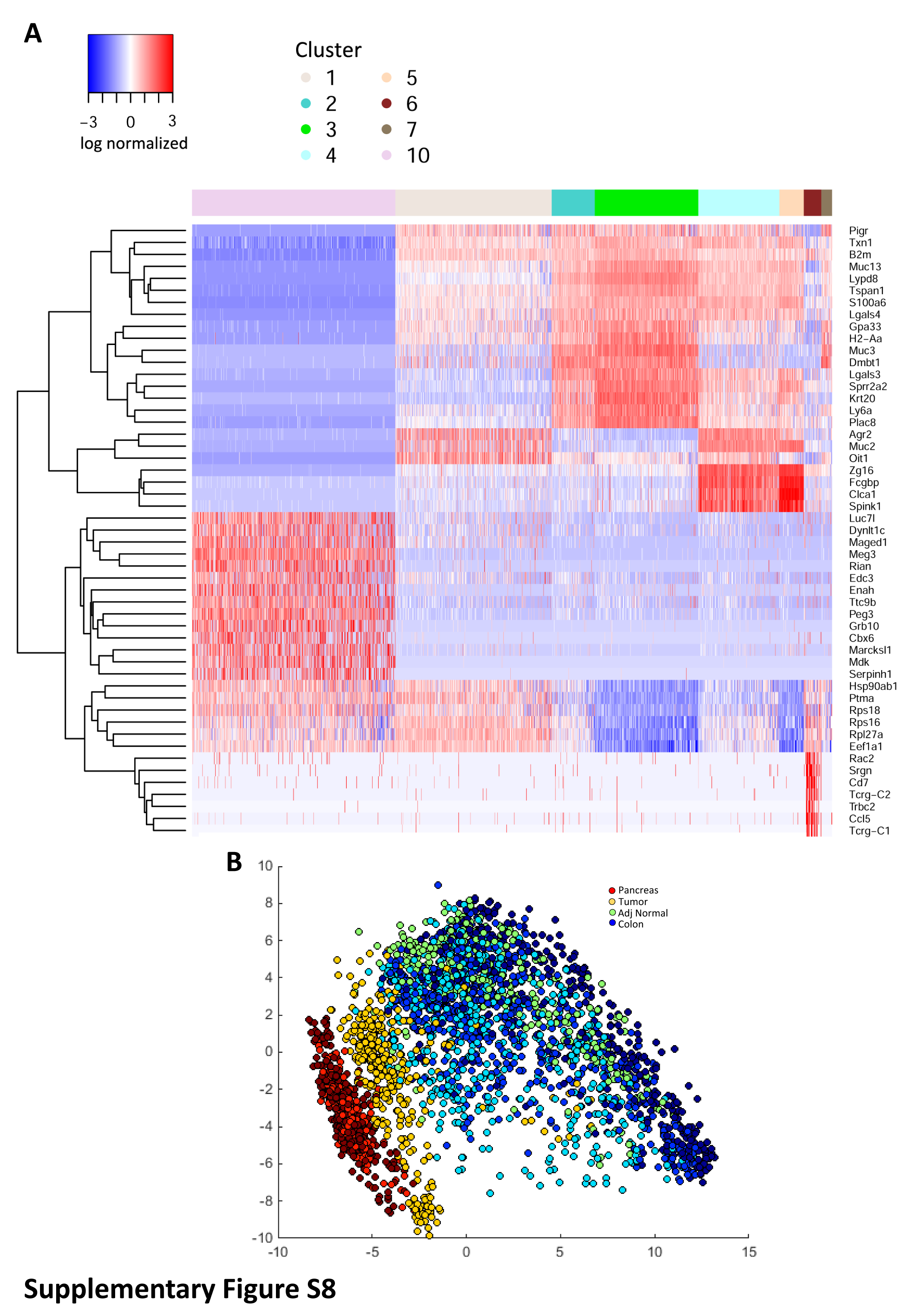

Supplement: S8 Fig — (A) Differential expressed gene identified by limma for each of the 10 groups in Fig 5. (B) PCA plot of multiple replicates of single-cell data from the pancreas, colonic tumor, adjacent normal colon, and normal colon analyzed together as in Fig 6A. (TIF) [file pbio.2006687.s008.tif]

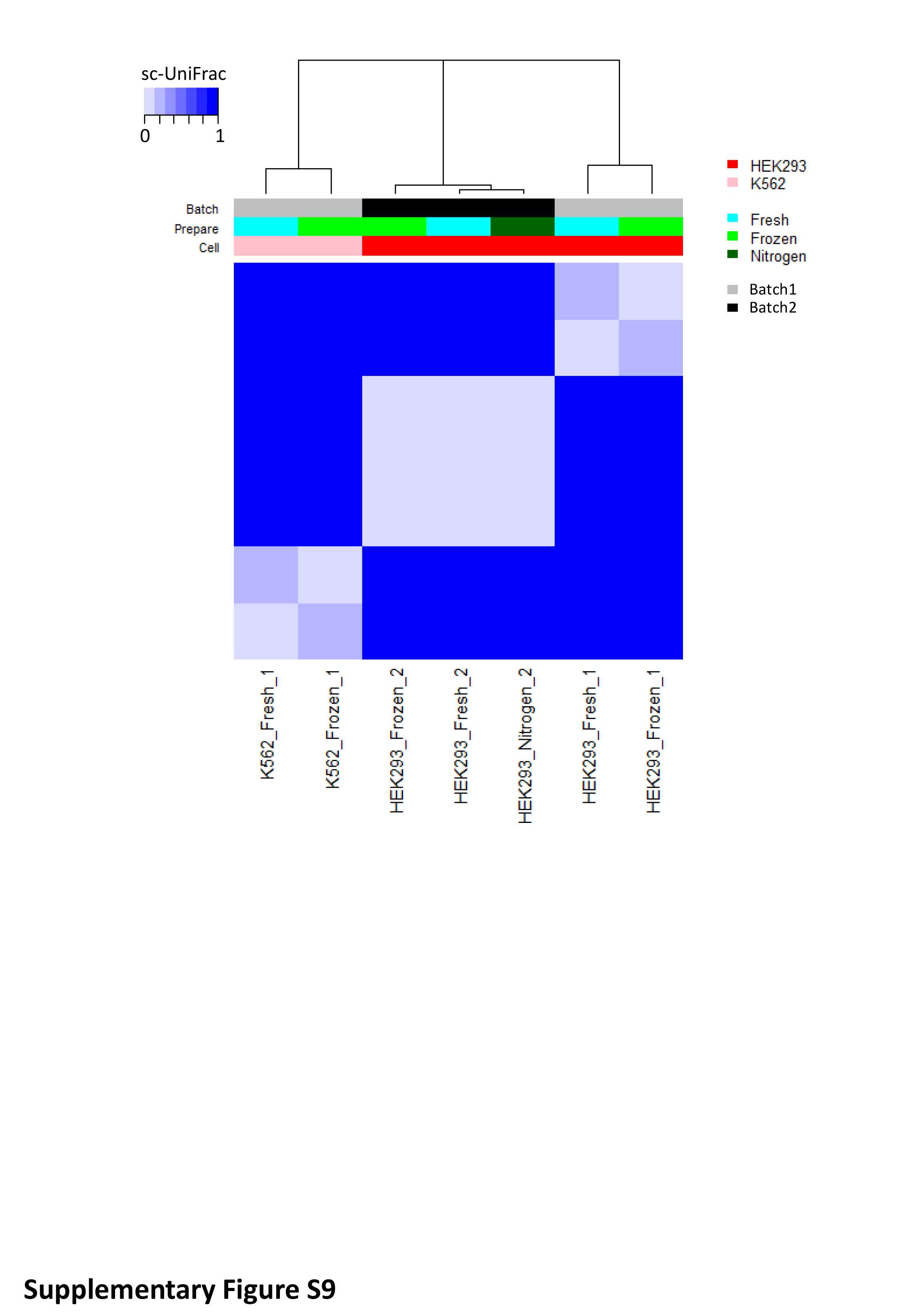

Supplement: S9 Fig — Hierarchical clustering by sc-UniFrac of scRNA-seq data from cell lines that are prepared differently (GSE85534) [35]. Heat depicts the sc-UniFrac distance between 2 samples. The results are consistent with the original study, which shows that the freezing process did not alter transcriptional profiles. In contrast, batch effects have a larger impact on the transcription profiles than the freezing process. (TIF) [file pbio.2006687.s009.tif]

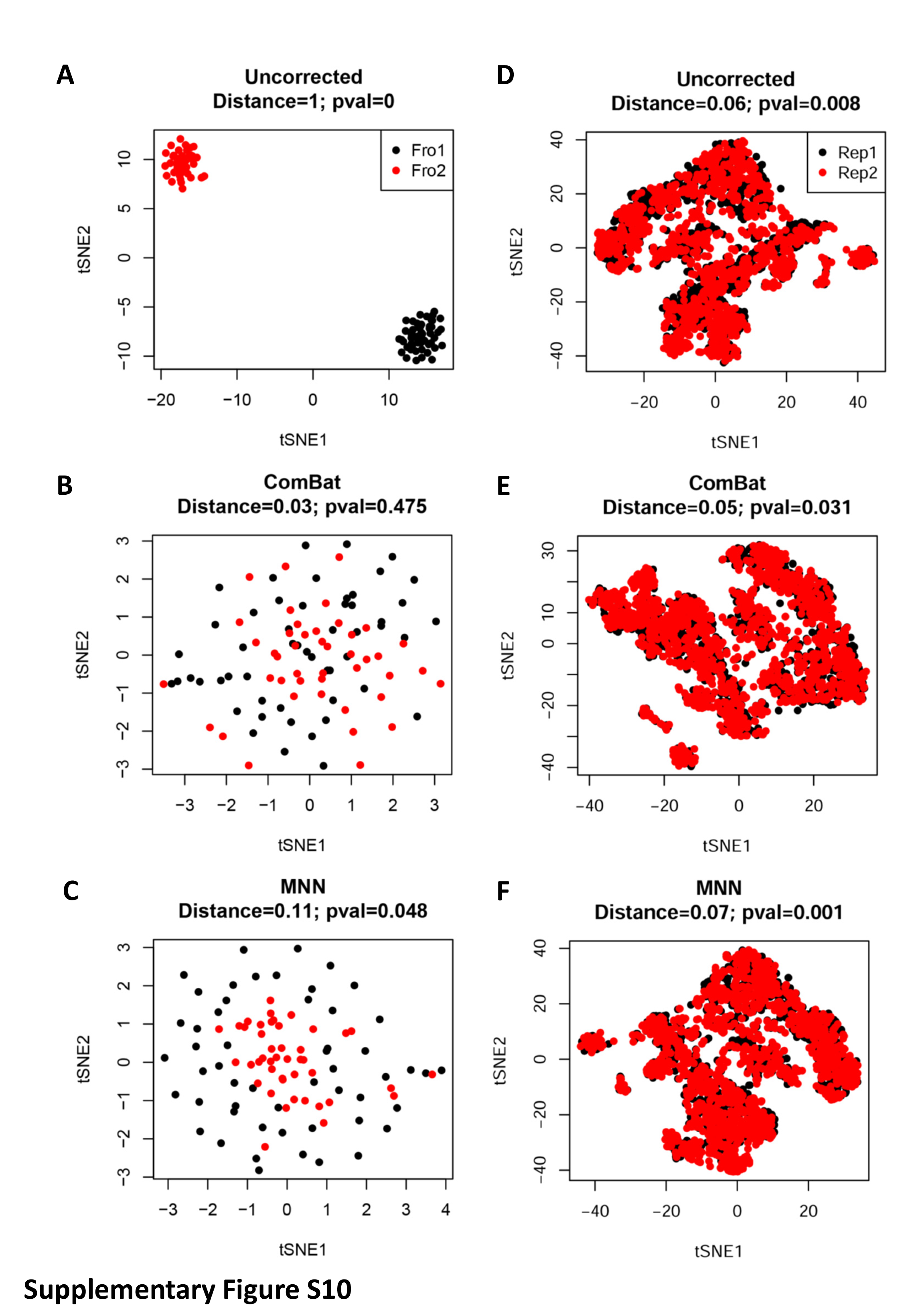

Supplement: S10 Fig — t-SNE analysis of scRNA-seq data from cell lines prepared from two batches (Frozen 1 and 2) [35] (A) uncorrected, (B) corrected by ComBat, and (C) corrected by MNN. t-SNE analysis of scRNA-seq data from the colonic mucosa from two technical replicates (Replicates 1 and 2). sc-UniFrac distance between the samples and p-value noted. (TIF) [file pbio.2006687.s010.tif]

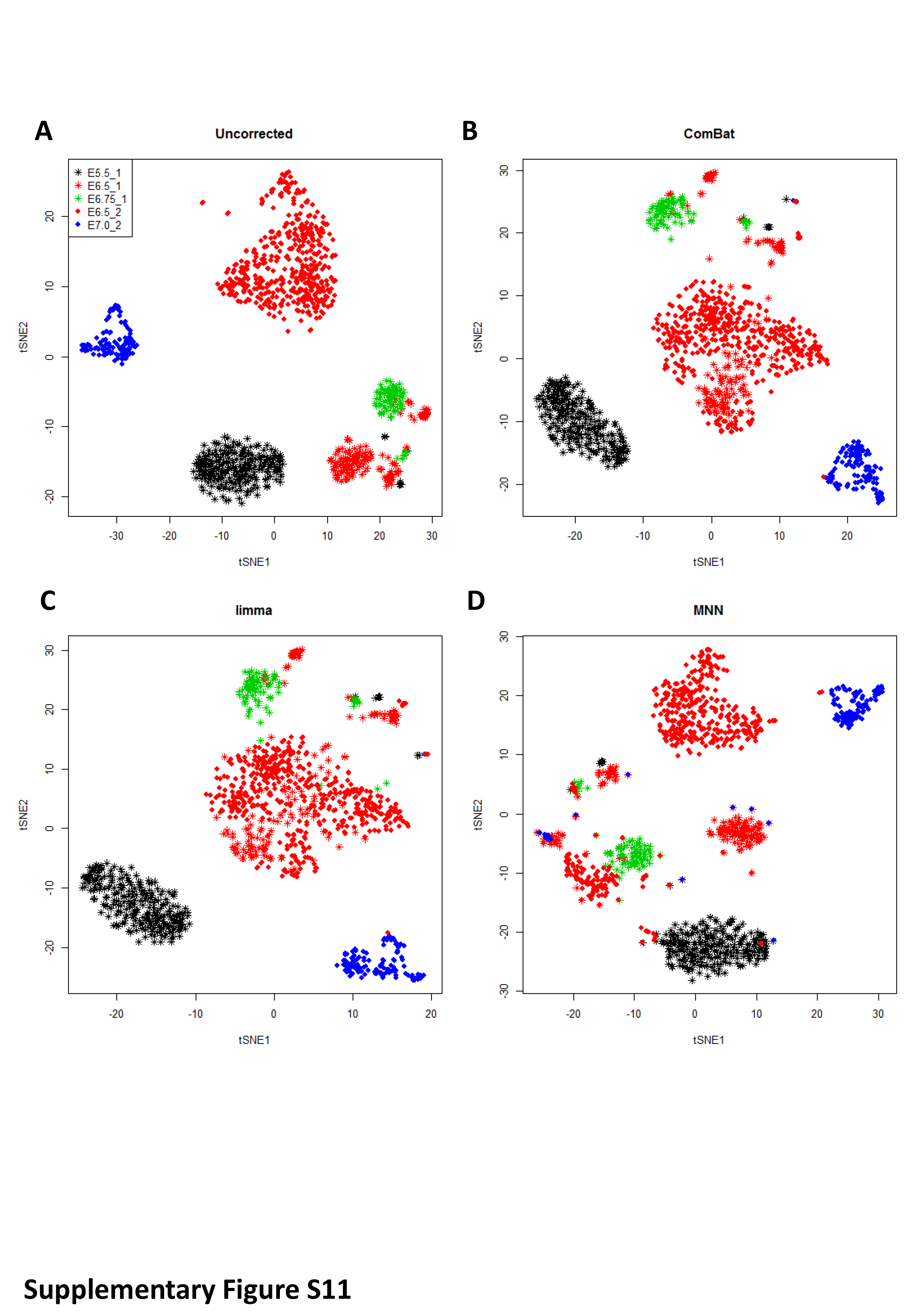

Supplement: S11 Fig — t-SNE analysis of scRNA-seq data depicting mouse gastrulation, with colors representing developmental time and shapes of data points representing the two studies [36,37]. For instance, all red data points should cluster together. Analysis performed on (A) uncorrected, (B) ComBat-corrected, (C) limma-corrected, and (D) MNN-corrected data. (TIF) [file pbio.2006687.s011.tif]
