# Supplementary material for: Quantitative assessment of cell population diversity in single-cell landscapes
Source: PLoS Biol. 2018 Oct 22;16(10):e2006687. doi: 10.1371/journal.pbio.2006687 (PMC6211764; doi:10.1371/journal.pbio.2006687)
Supplement: S1 Report — (PDF) [file pbio.2006687.s016.pdf]

scUnifrac Report

Contents

1 Overview **1**

2 Results Summary **1**

3 Subpopulation View **3**

3.1 The cell subpopulation 10 4
3.2 The cell subpopulation 1 6
3.3 The cell subpopulation 3 8
3.4 The cell subpopulation 4 10
3.5 The cell subpopulation 2 12
3.6 The cell subpopulation 5 14
3.7 The cell subpopulation 6 16
3.8 The cell subpopulation 7 18

4 Session Info **19**

1 Overview

scUnifrac is to quantify cell subpopulation diversity between two single-cell transcriptome profiles, which calculates the distance, estimates the statistical significance of the distance, identifies the subpopulations that are significant different between two profiles, finds genes that mark subpopulations, and predicts cell types of subpopulations. If two single-cell RNA-seq profiles have identical cell subpopulation structures (the same subpopulations with the same proportion), the distance is zero, whereas, the distance is one if completely different subpopulations.

- ***Results Summary*** - The distance, the statistical significance (p-value), the subpopulations that are different in two profiles, and phylogenetic tree plot and tSNE plot to show the subpopulation structures.
- ***Subpopulation view*** - heatmap of gene expression that mark the statistical significant subpopulations, and predicted cell types of these subpopulations based on Mouse Cell Atlas [Han et al., 2018, Cell, 1091-1107] if scRNA-seq are derived from mouse samples.

2 Results Summary

Subpopulation structure:

Table 1: The number of cells in each subpopulation in two scRNA-seq profiles

Subpopulation	1	2	3	4	5	6	7	8	9	10
Colon	411	113	272	213	64	46	28	7	8	0
Pan	0	0	0	0	0	0	0	0	0	534

Table 2: Significantly different subpopulations and their proportion difference between two scRNA-seq profiles

Subpopulation	10	1	3	4	2	5	6	7
Proportion difference	1.00	0.35	0.23	0.18	0.10	0.06	0.04	0.02

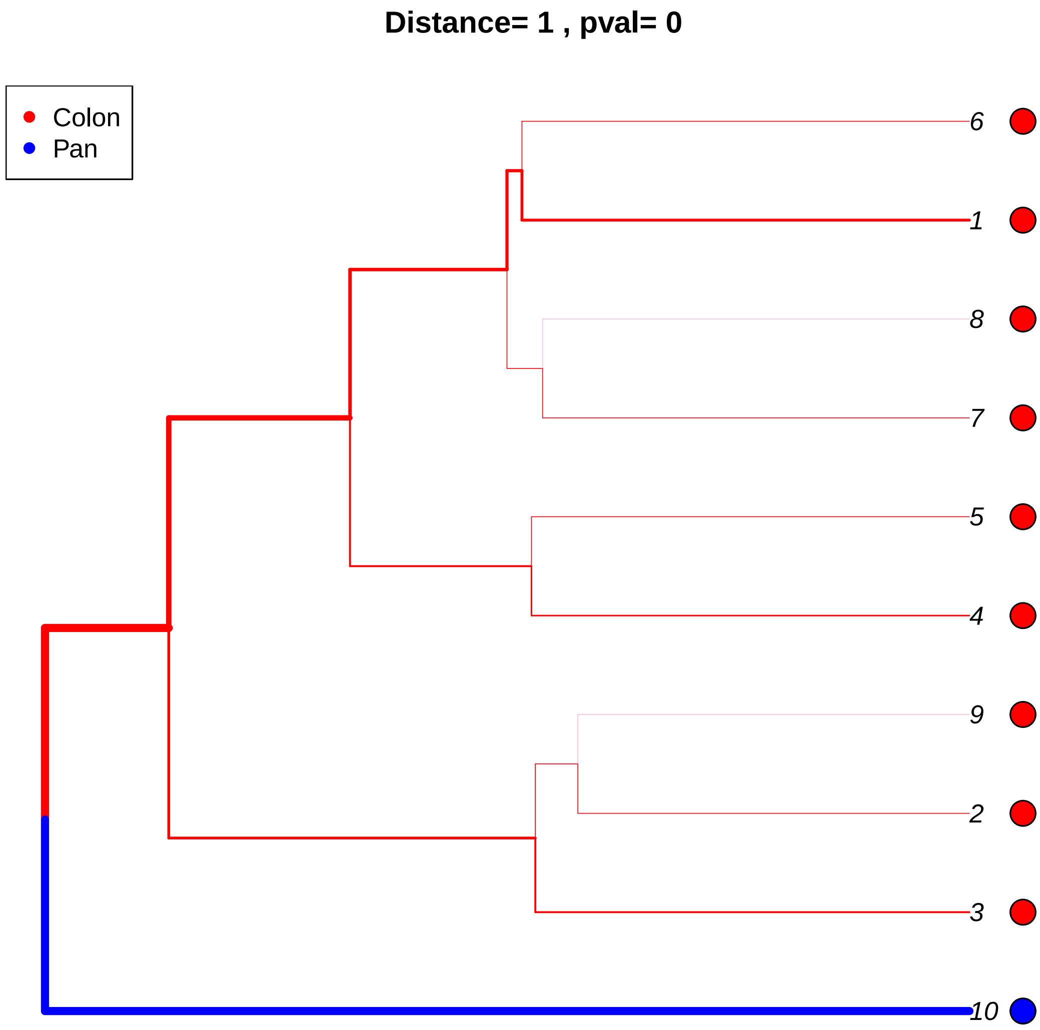

Figure 1: Phylogenetic tree plot of subpopulation structures. Cell subpopulations that are enriched in the first scRNA-seq profile labeled as red, while subpopulations enriched in the second profile are labeled as blue. The line width is proportion to the enrichment. Thicker lines suggest more enriched in one profile. Cell subpopulations that are similar in both samples are labeled as black.

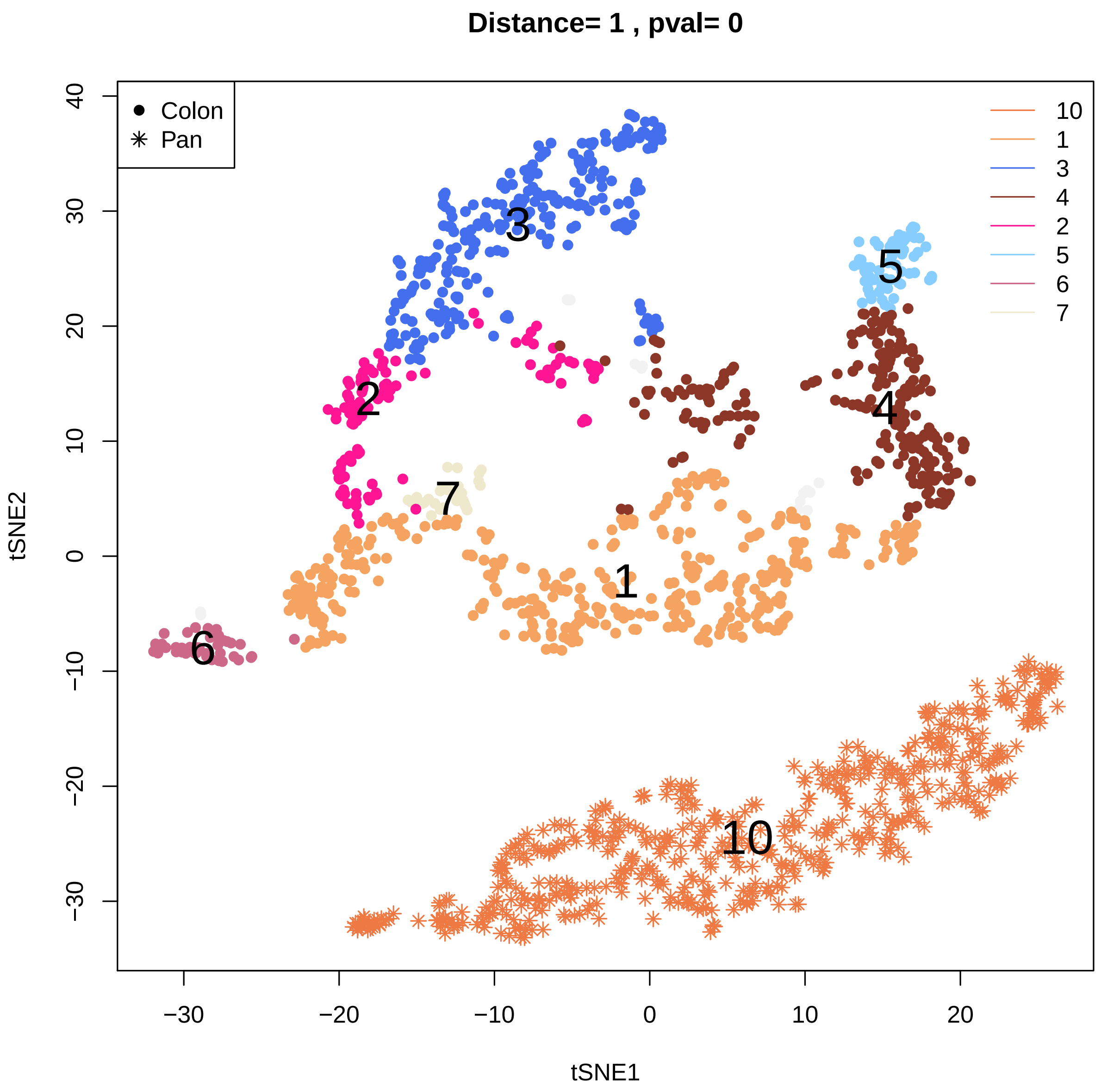

Figure 2: tSNE plot of subpopulation structures. Each subpopulation that are different between two profiles are denoted as different colors, and subpopulations that are similar between two profiles are denotes as gray. Cells from the first profile are denoted by circle, while cell from the second profile are denoted by star.

3 Subpopulation View

3.1 The cell subpopulation 10

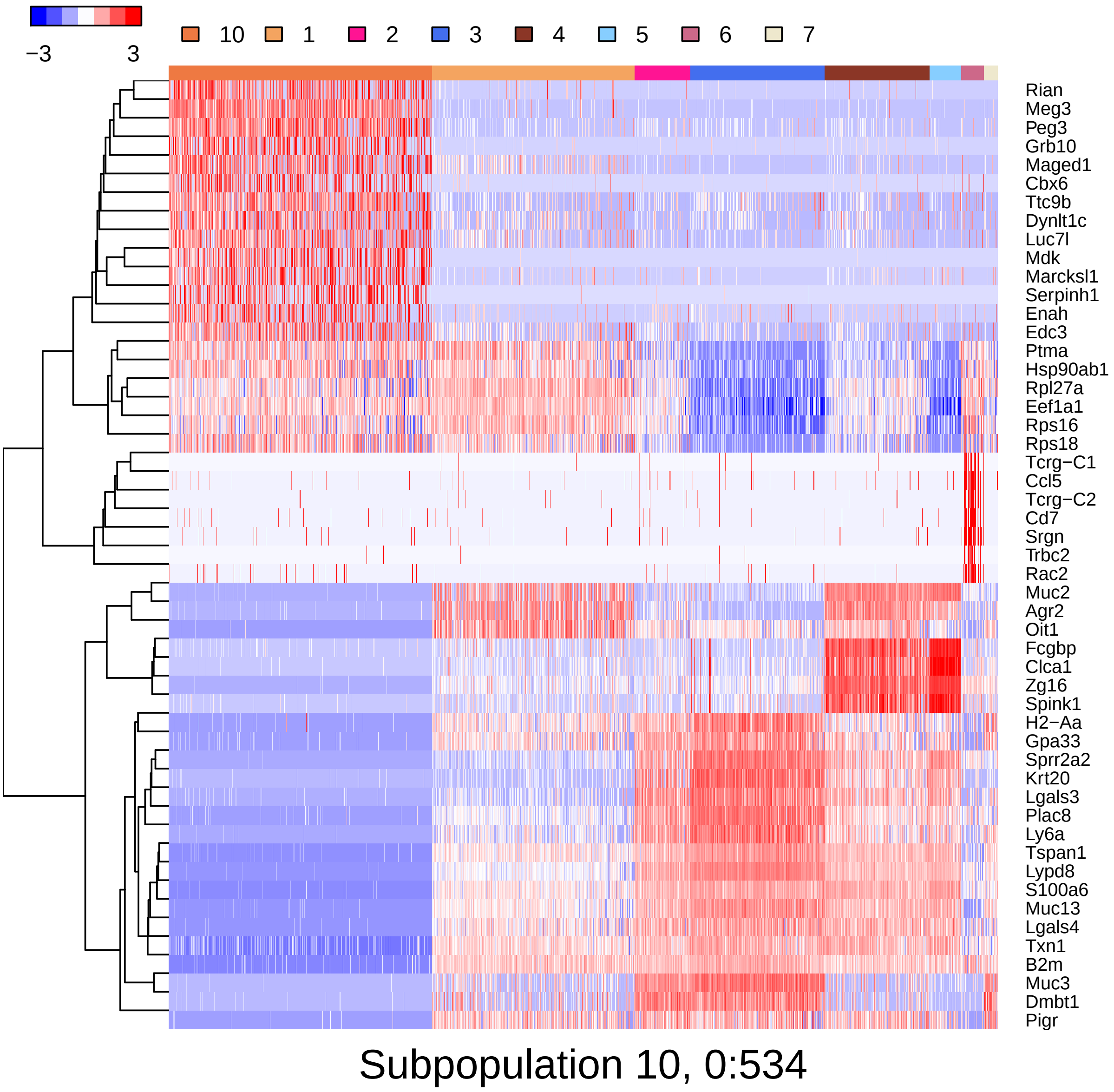

Figure 3: Heatmap of gene expressions that mark the cell subpopulation 10

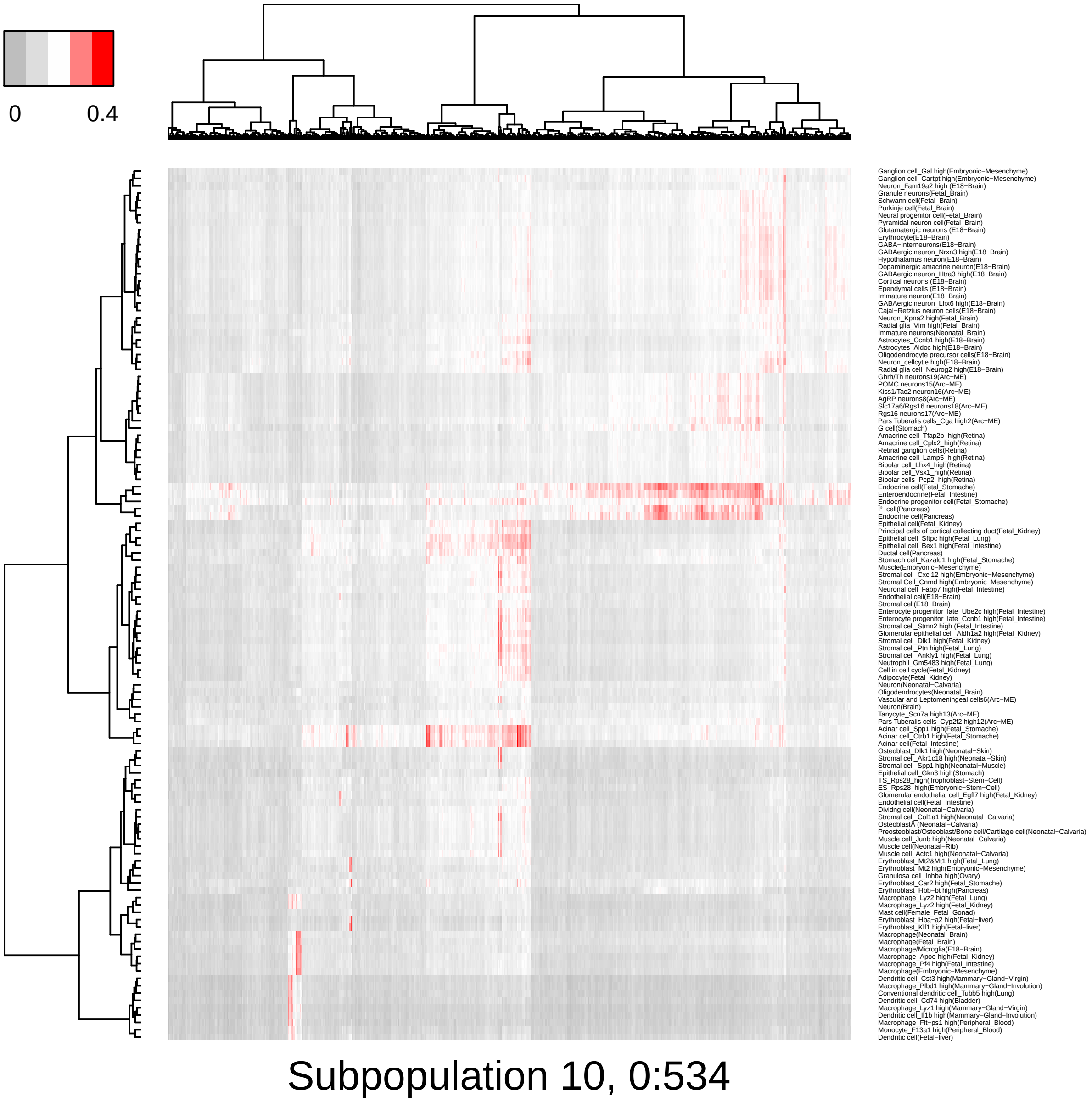

Figure 4: Predicted cell types of the cell subpopulation 10

3.2 The cell subpopulation 1

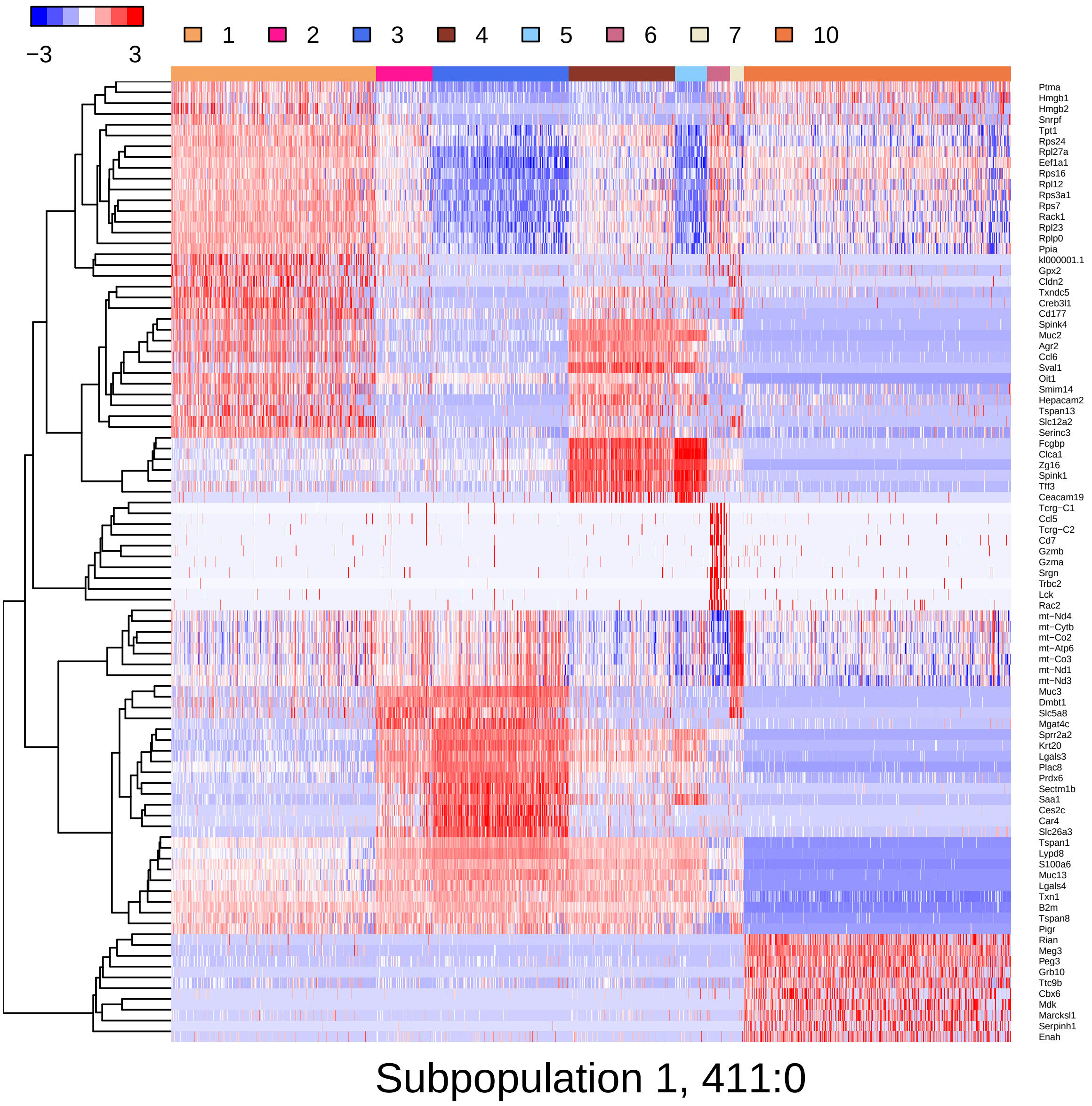

Figure 5: Heatmap of gene expressions that mark the cell subpopulation 1

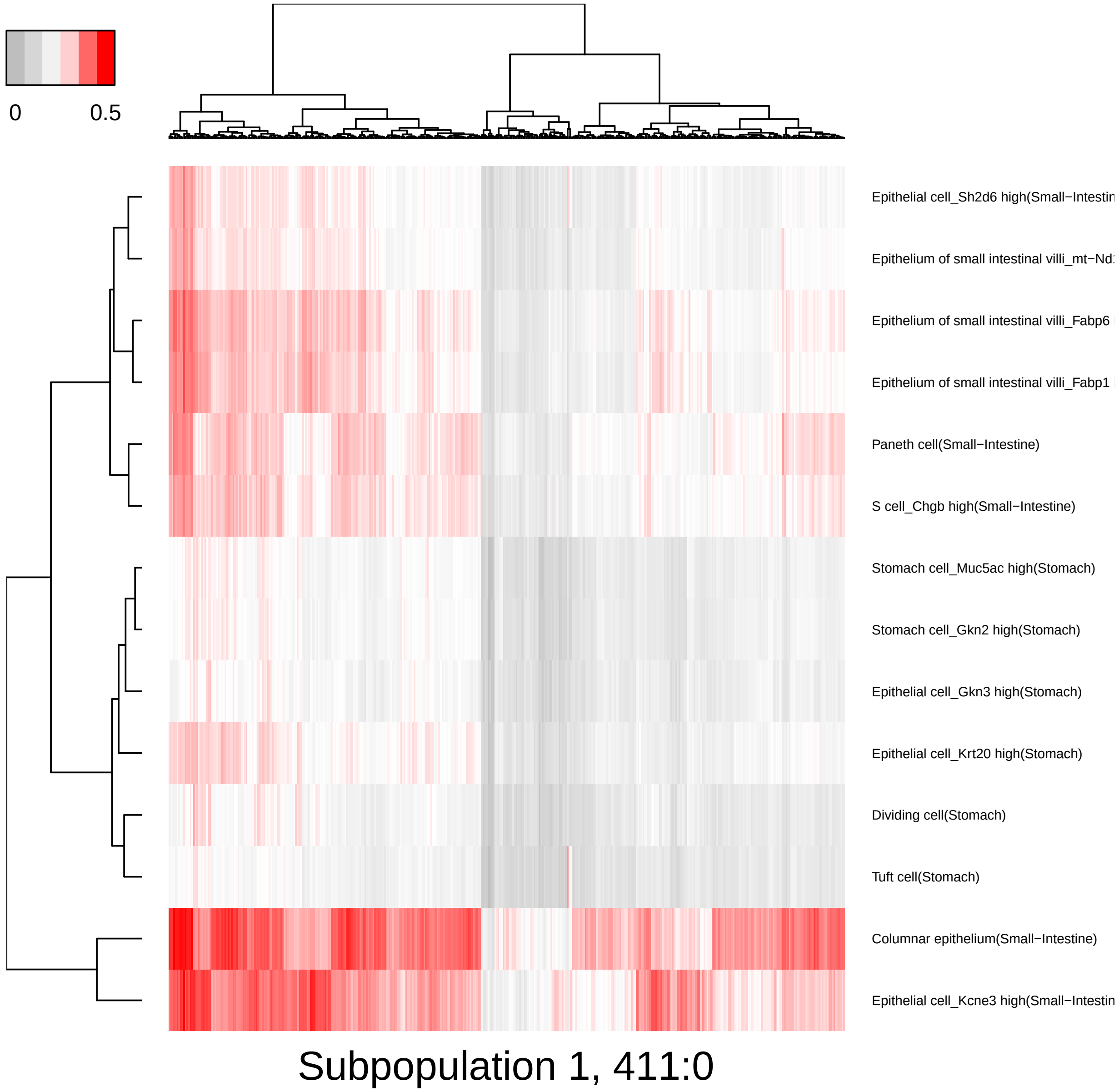

Figure 6: Predicted cell types of the cell subpopulation 1

3.3 The cell subpopulation 3

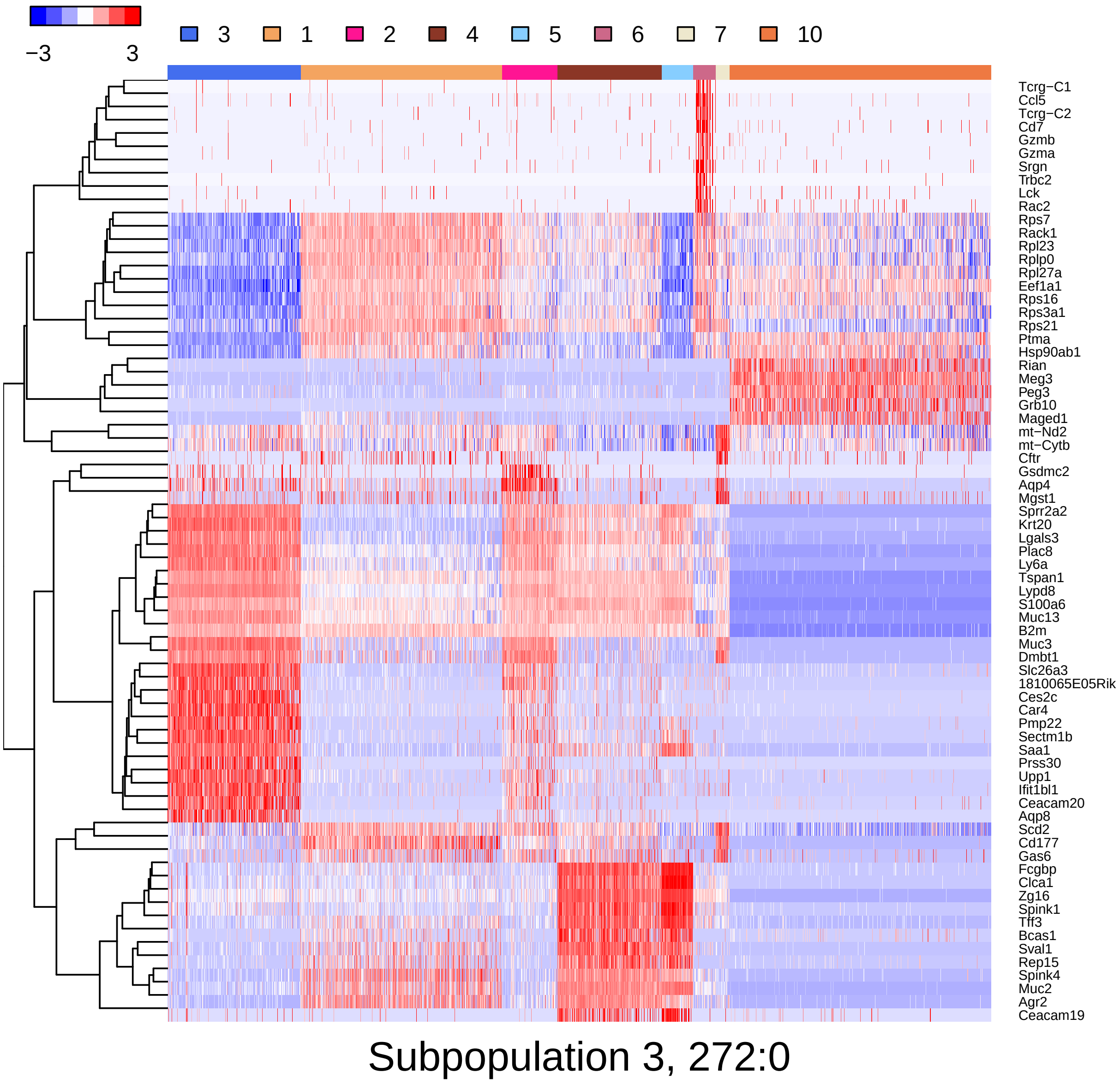

Figure 7: Heatmap of gene expressions that mark the cell subpopulation 3

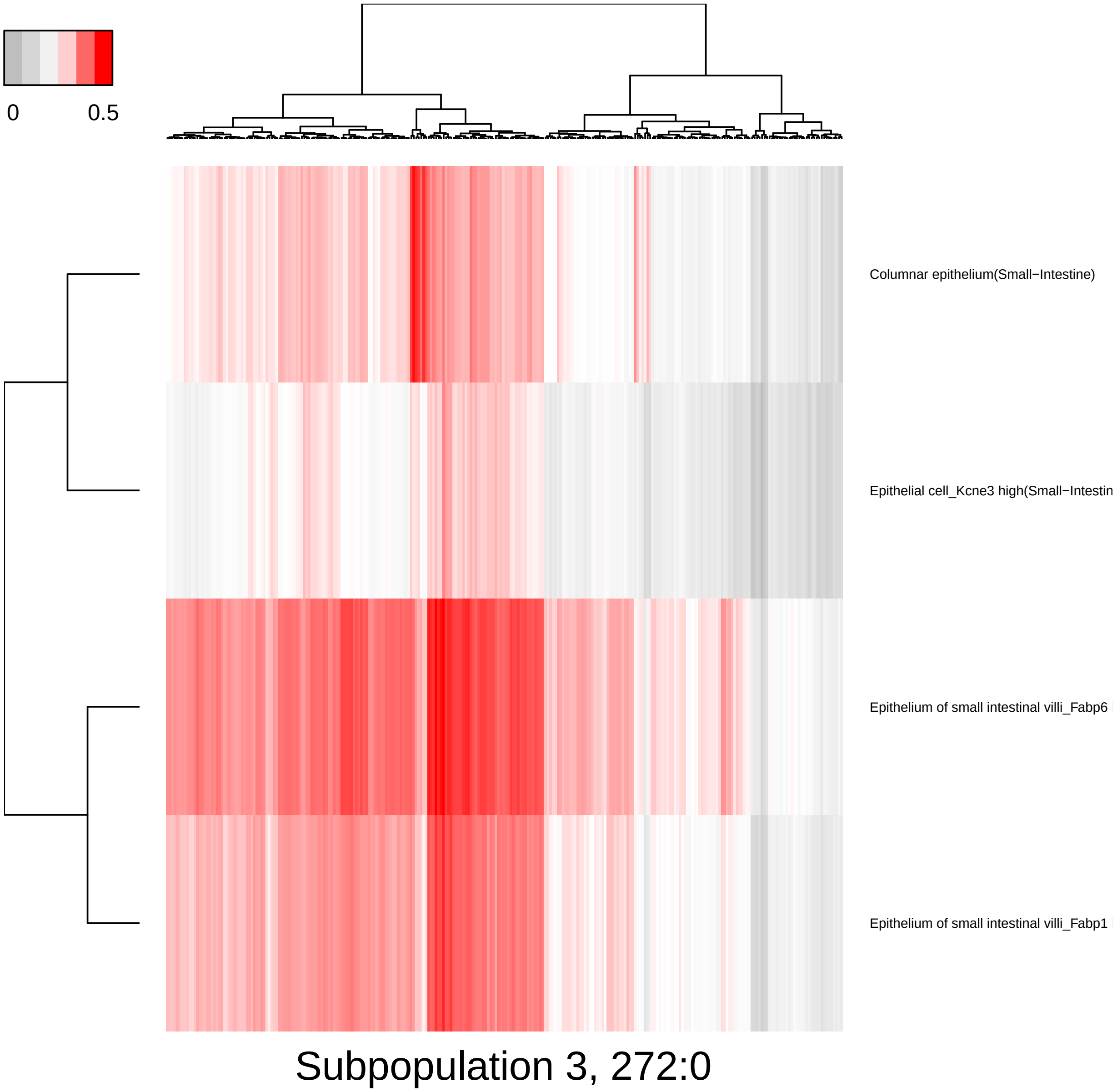

Figure 8: Predicted cell types of the cell subpopulation 3

3.4 The cell subpopulation 4

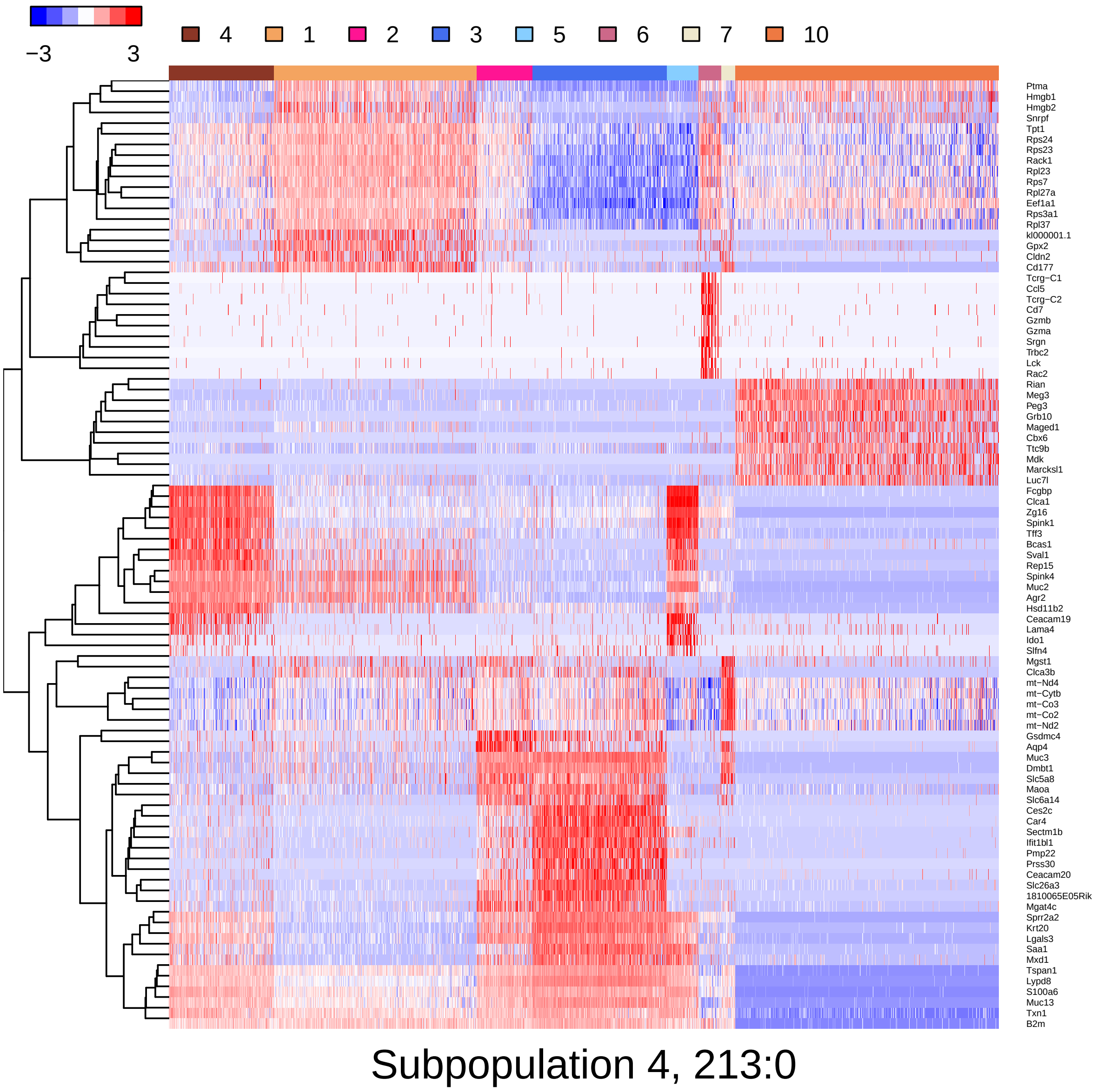

Figure 9: Heatmap of gene expressions that mark the cell subpopulation 4

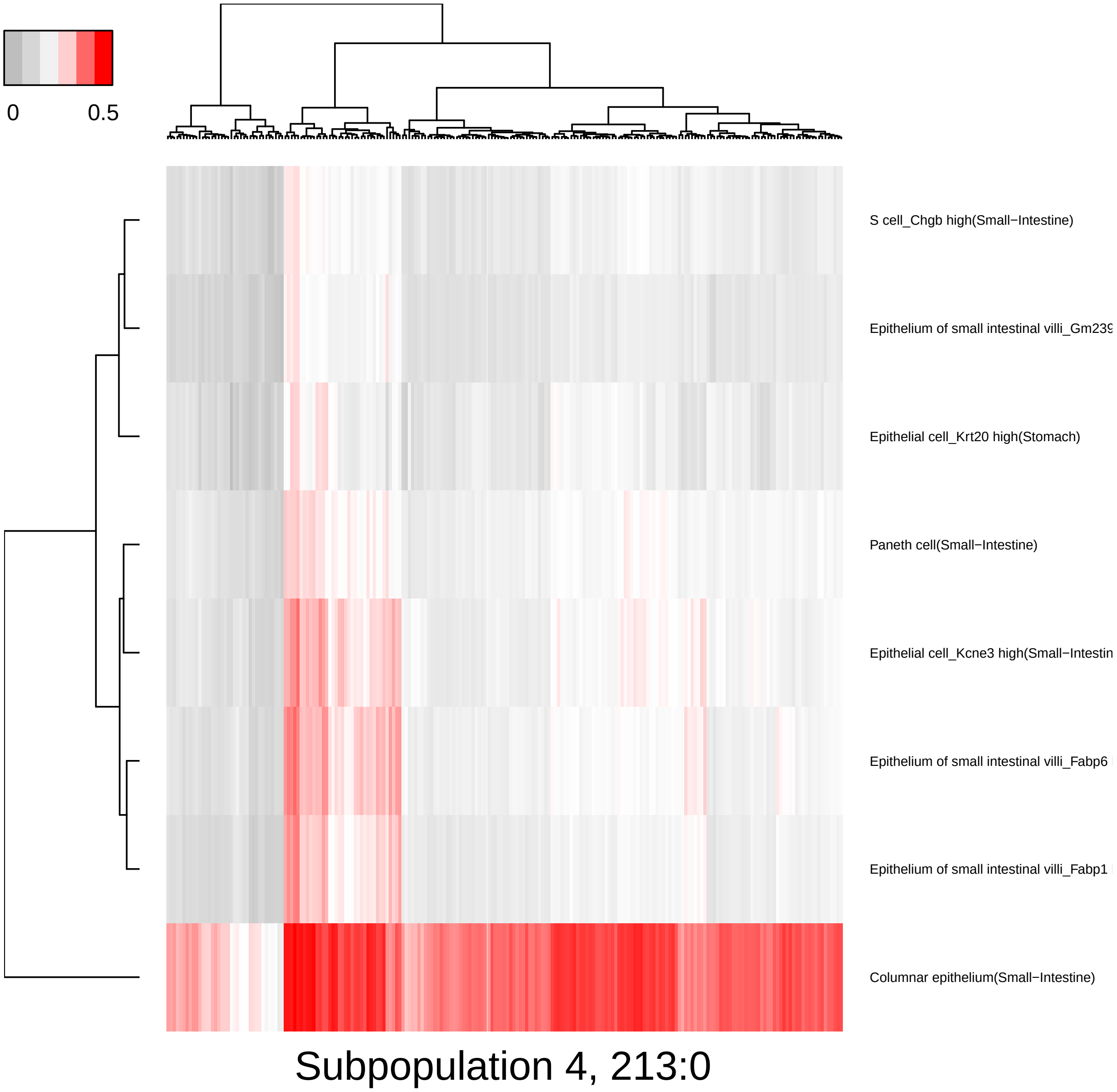

Figure 10: Predicted cell types of the cell subpopulation 4

3.5 The cell subpopulation 2

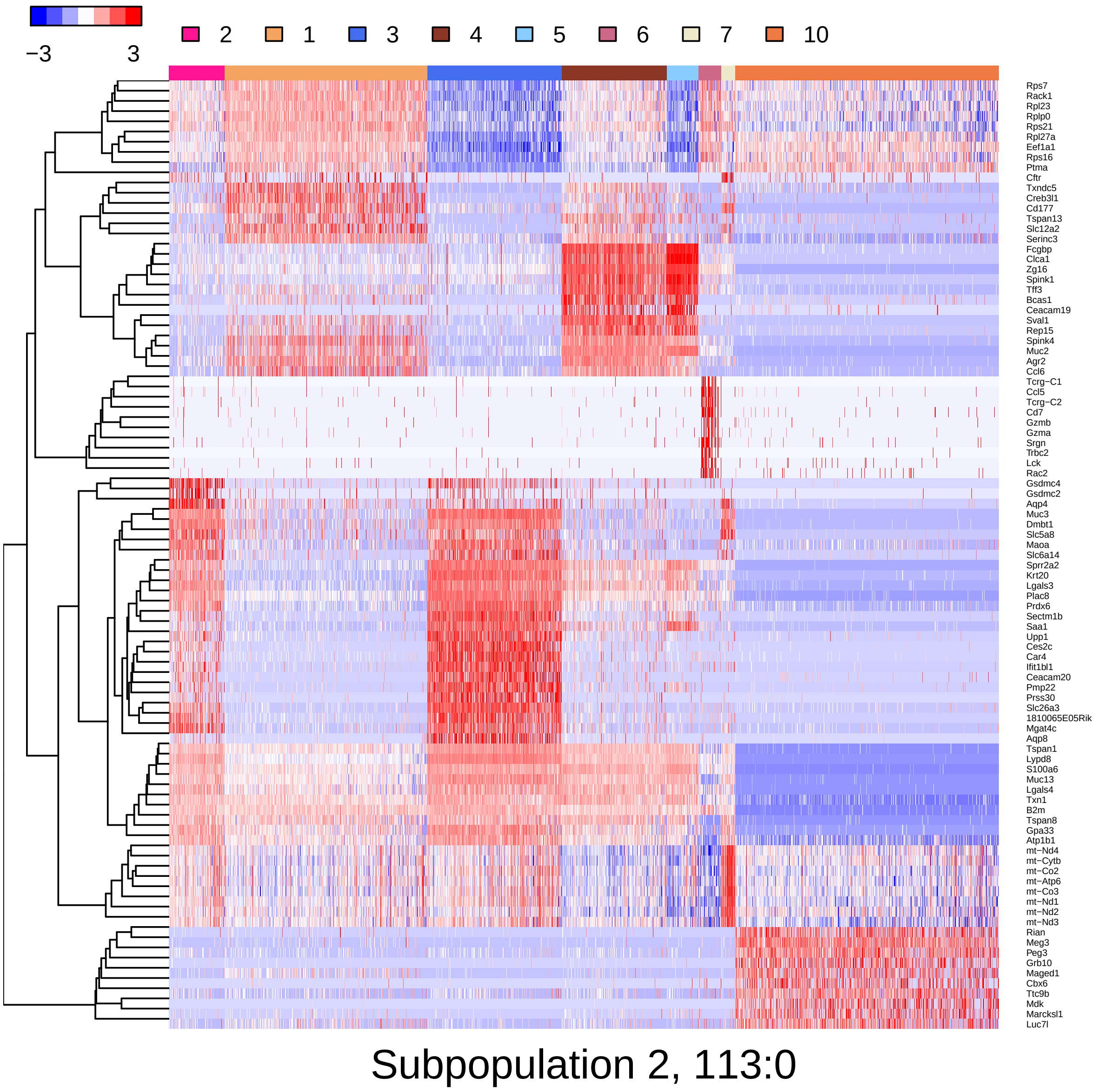

Figure 11: Heatmap of gene expressions that mark the cell subpopulation 2

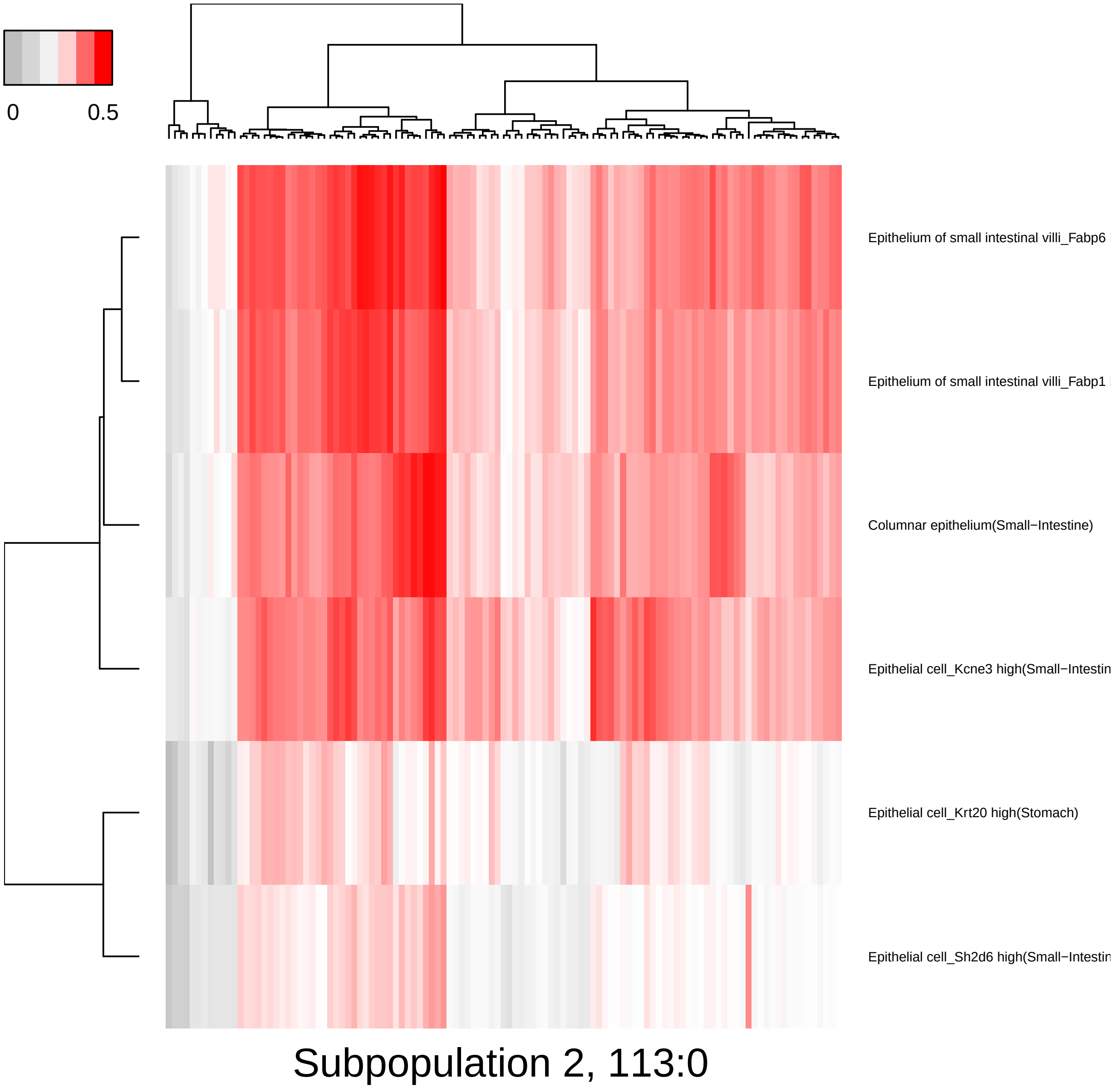

Figure 12: Predicted cell types of the cell subpopulation 2

3.6 The cell subpopulation 5

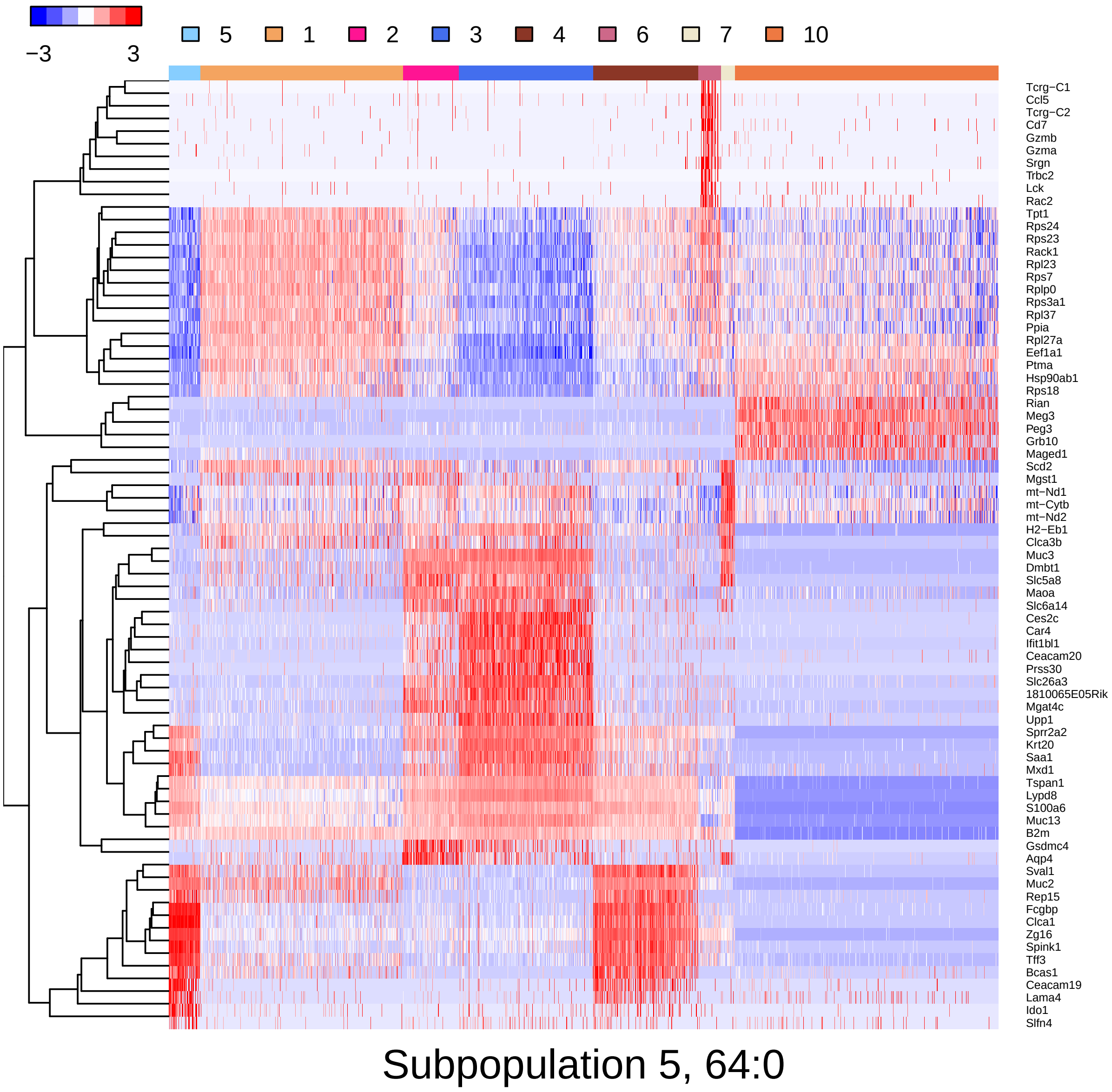

Figure 13: Heatmap of gene expressions that mark the cell subpopulation 5

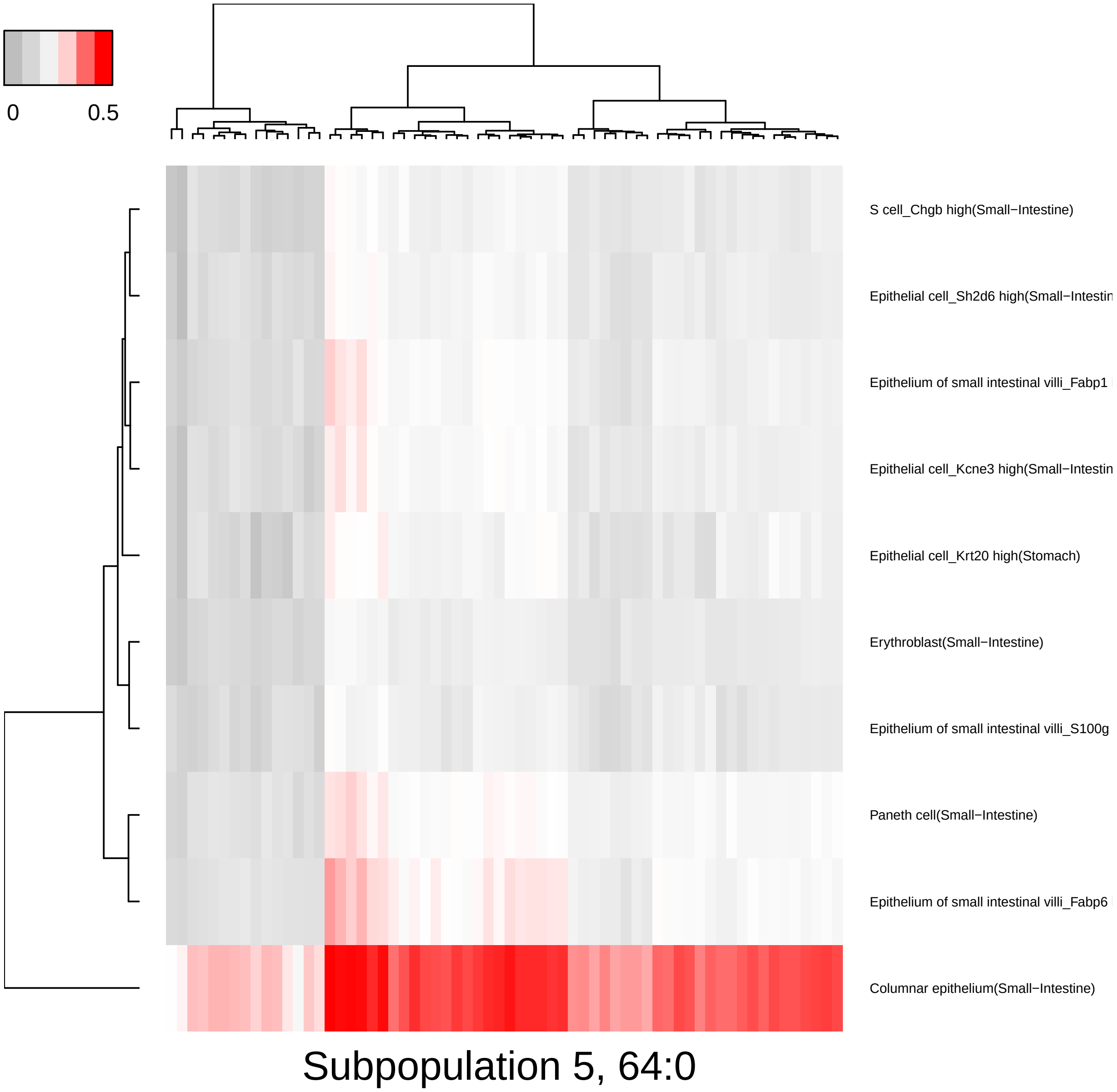

Figure 14: Predicted cell types of the cell subpopulation 5

3.7 The cell subpopulation 6

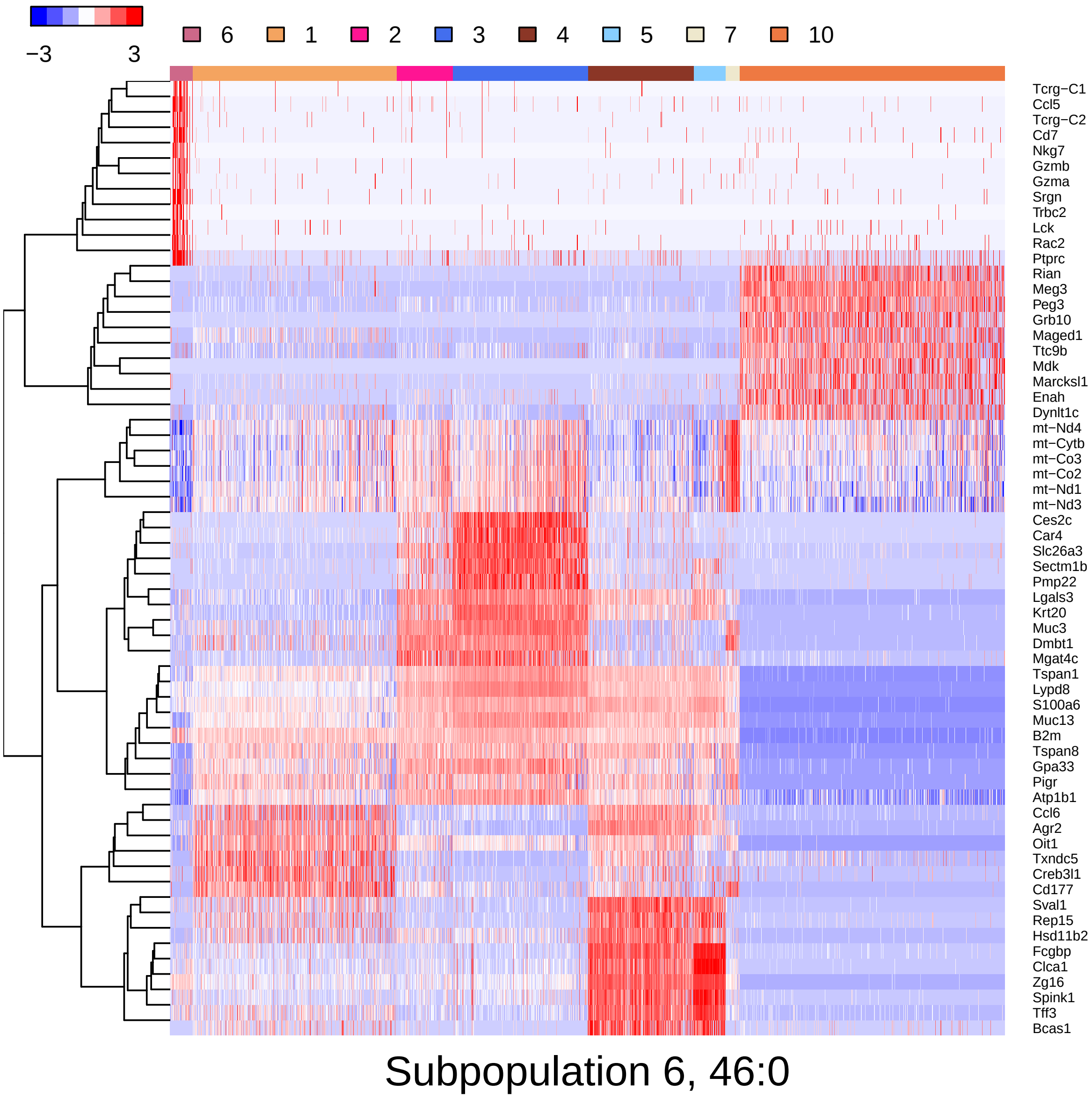

Figure 15: Heatmap of gene expressions that mark the cell subpopulation 6

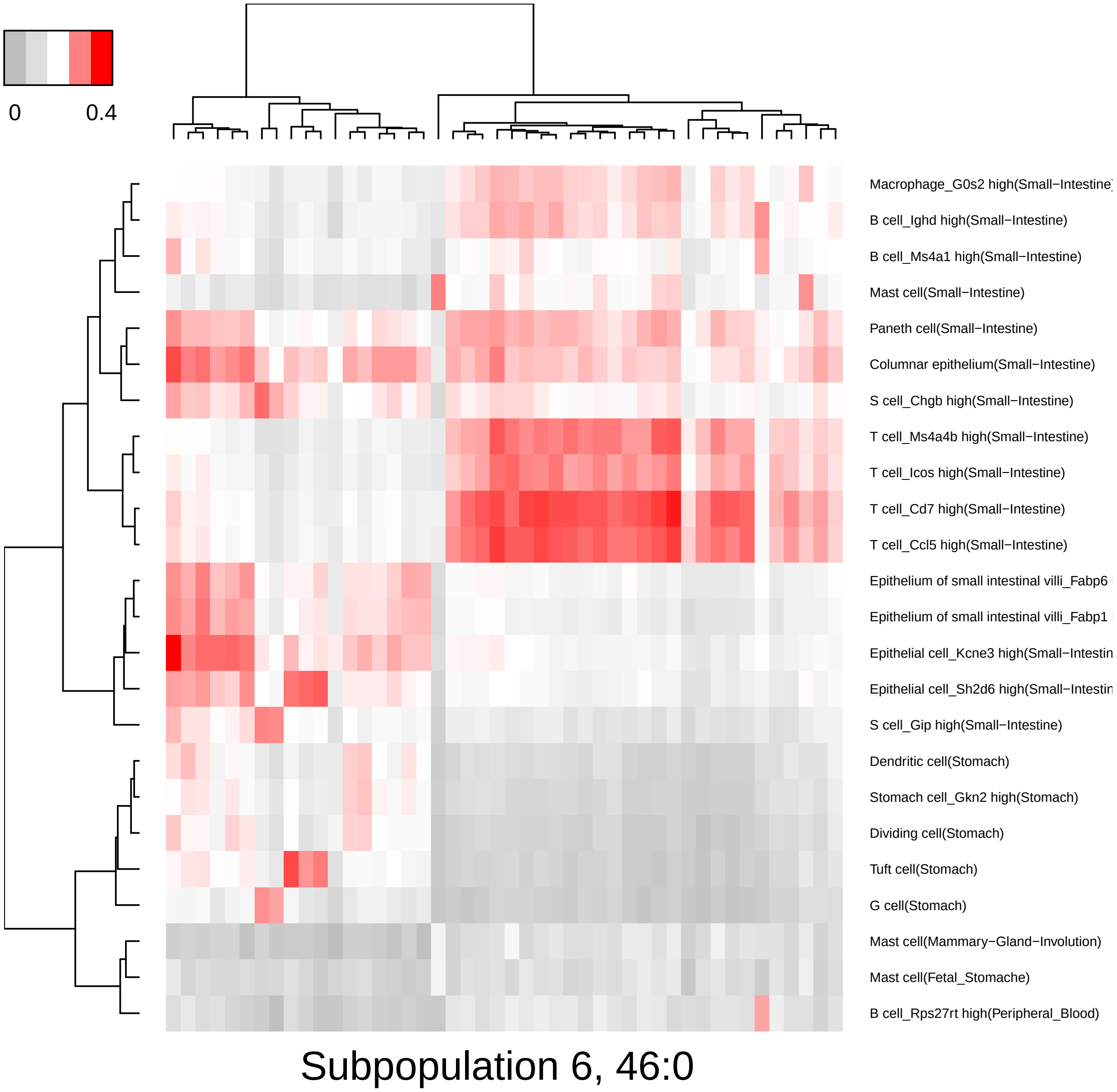

Figure 16: Predicted cell types of the cell subpopulation 6

3.8 The cell subpopulation 7

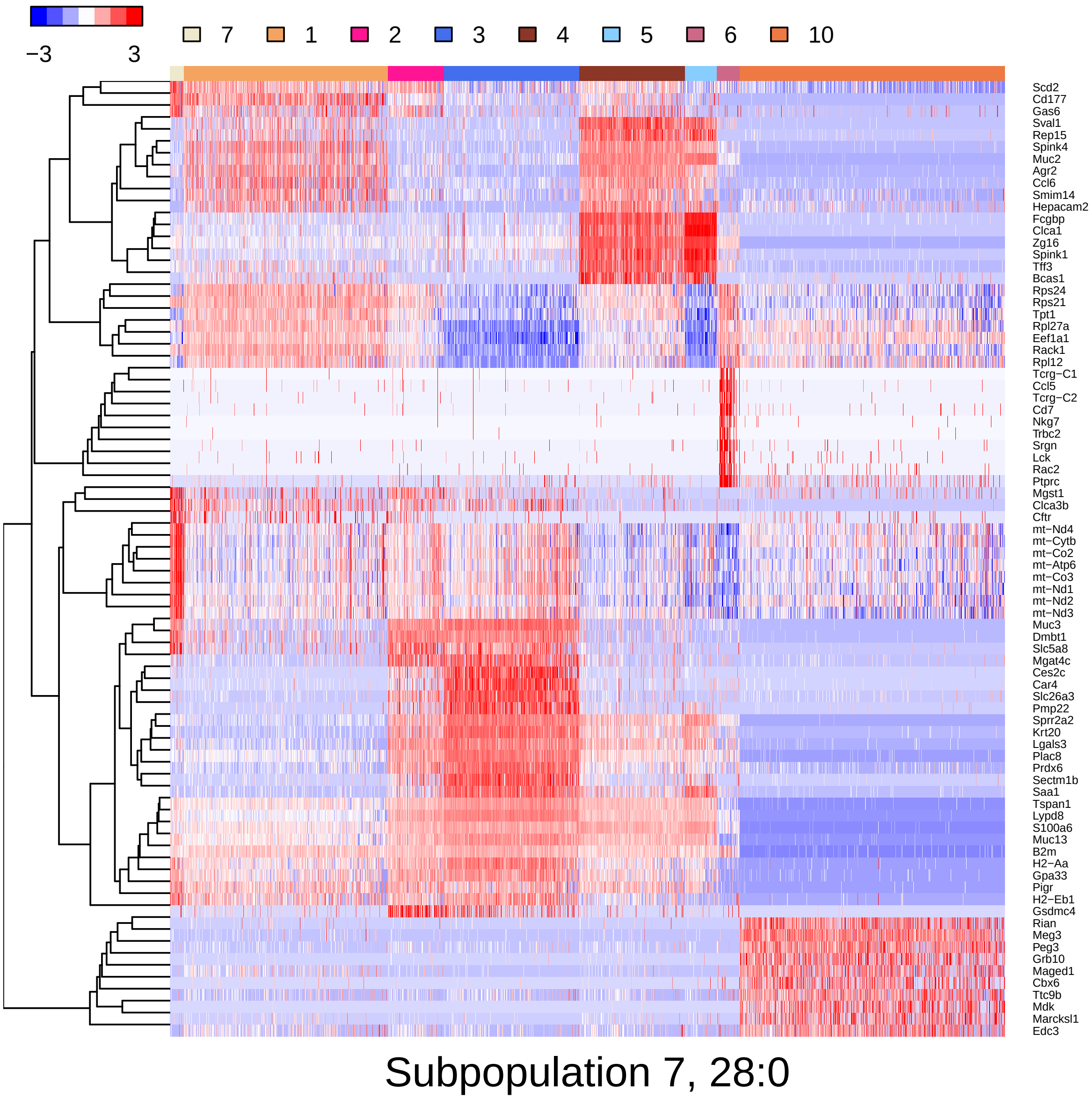

Figure 17: Heatmap of gene expressions that mark the cell subpopulation 7

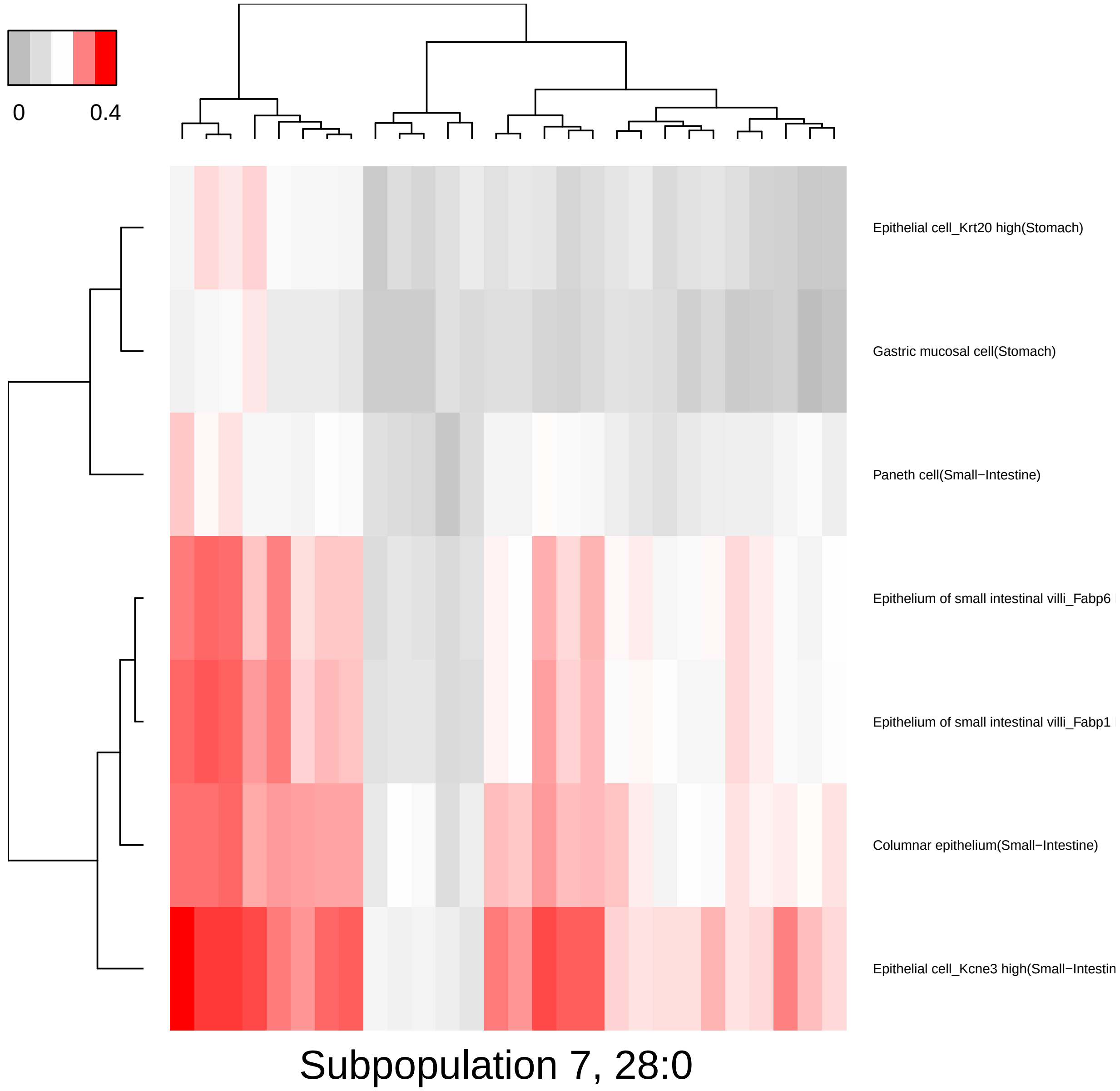

Figure 18: Predicted cell types of the cell subpopulation 7

4 Session Info

```
devtools::session_info()
```

```
## setting  value
## version  R version 3.4.4 (2018-03-15)
## system   x86_64, mingw32
## ui       RStudio (1.1.442)
## language (EN)
## collate  English_United States.1252
```

```
##  tz       America/Chicago
##  date     2018-05-14
##
##  package     * version   date       source
##  ape         * 5.1       2018-04-04 CRAN (R 3.4.4)
##  backports     1.1.2     2017-12-13 CRAN (R 3.4.3)
##  base        * 3.4.4     2018-03-15 local
##  bookdown      0.7       2018-02-18 CRAN (R 3.4.4)
##  cluster       2.0.6     2017-03-16 CRAN (R 3.4.4)
##  colorspace    1.3-2     2016-12-14 CRAN (R 3.4.4)
##  compiler      3.4.4     2018-03-15 local
##  curl          3.1       2017-12-12 CRAN (R 3.4.3)
##  datasets    * 3.4.4     2018-03-15 local
##  devtools    * 1.13.5    2018-02-18 CRAN (R 3.4.3)
##  digest        0.6.15    2018-01-28 CRAN (R 3.4.3)
##  evaluate      0.10.1    2017-06-24 CRAN (R 3.4.4)
##  git2r         0.21.0    2018-01-04 CRAN (R 3.4.4)
##  graphics    * 3.4.4     2018-03-15 local
##  grDevices   * 3.4.4     2018-03-15 local
##  grid          3.4.4     2018-03-15 local
##  GUniFrac    * 1.1       2018-02-12 CRAN (R 3.4.4)
##  highr         0.6       2016-05-09 CRAN (R 3.4.4)
##  hms           0.4.2     2018-03-10 CRAN (R 3.4.4)
##  htmltools     0.3.6     2017-04-28 CRAN (R 3.4.4)
##  httpuv        1.3.6.2   2018-03-02 CRAN (R 3.4.4)
##  httr          1.3.1     2017-08-20 CRAN (R 3.4.4)
##  kableExtra  * 0.8.0     2018-04-05 CRAN (R 3.4.4)
##  knitr       * 1.20      2018-02-20 CRAN (R 3.4.4)
##  lattice     * 0.20-35   2017-03-25 CRAN (R 3.4.4)
##  limma       * 3.34.9    2018-02-22 Bioconductor
##  magrittr      1.5       2014-11-22 CRAN (R 3.4.4)
##  MASS          7.3-49    2018-02-23 CRAN (R 3.4.4)
##  Matrix        1.2-12    2017-11-16 CRAN (R 3.4.4)
##  matrixStats * 0.53.1    2018-02-11 CRAN (R 3.4.4)
##  memoise       1.1.0     2017-04-21 CRAN (R 3.4.4)
##  methods     * 3.4.4     2018-03-15 local
##  mgcv          1.8-23    2018-01-15 CRAN (R 3.4.4)
##  mime          0.5       2016-07-07 CRAN (R 3.4.1)
##  miniUI        0.1.1     2016-01-15 CRAN (R 3.4.4)
##  munsell       0.4.3     2016-02-13 CRAN (R 3.4.4)
##  nlme          3.1-131.1 2018-02-16 CRAN (R 3.4.4)
##  parallel      3.4.4     2018-03-15 local
##  permute     * 0.9-4     2016-09-09 CRAN (R 3.4.4)
##  pillar        1.2.1     2018-02-27 CRAN (R 3.4.4)
##  pkgconfig     2.0.1     2017-03-21 CRAN (R 3.4.4)
##  plyr          1.8.4     2016-06-08 CRAN (R 3.4.4)
##  questionr     0.6.2     2017-11-01 CRAN (R 3.4.4)
##  R.methodsS3 * 1.7.1     2016-02-16 CRAN (R 3.4.1)
##  R.oo        * 1.21.0    2016-11-01 CRAN (R 3.4.1)
##  R.utils     * 2.6.0     2017-11-05 CRAN (R 3.4.4)
##  R6            2.2.2     2017-06-17 CRAN (R 3.4.4)
##  Rcpp          0.12.16   2018-03-13 CRAN (R 3.4.4)
##  readr         1.1.1     2017-05-16 CRAN (R 3.4.4)
##  rlang         0.2.0     2018-02-20 CRAN (R 3.4.4)
```

```
##  rmarkdown     1.9       2018-03-01 CRAN (R 3.4.4)
##  rmdformats  * 0.3.3     2017-05-18 CRAN (R 3.4.4)
##  rprojroot     1.3-2     2018-01-03 CRAN (R 3.4.4)
##  rstudioapi    0.7       2017-09-07 CRAN (R 3.4.4)
##  Rtsne       * 0.13      2017-04-14 CRAN (R 3.4.4)
##  rvest         0.3.2     2016-06-17 CRAN (R 3.4.4)
##  scales        0.5.0     2017-08-24 CRAN (R 3.4.4)
##  scUnifrac   * 0.9.3     2018-05-14 Github (liuqivandy/scUnifrac@fb6fd0d)
##  shiny         1.0.5     2017-08-23 CRAN (R 3.4.4)
##  splines       3.4.4     2018-03-15 local
##  statmod       1.4.30    2017-06-18 CRAN (R 3.4.4)
##  stats       * 3.4.4     2018-03-15 local
##  stringi       1.1.7     2018-03-12 CRAN (R 3.4.4)
##  stringr       1.3.0     2018-02-19 CRAN (R 3.4.4)
##  tibble        1.4.2     2018-01-22 CRAN (R 3.4.4)
##  tools         3.4.4     2018-03-15 local
##  utils       * 3.4.4     2018-03-15 local
##  vegan       * 2.5-1     2018-04-14 CRAN (R 3.4.4)
##  viridisLite   0.3.0     2018-02-01 CRAN (R 3.4.4)
##  withr         2.1.2     2018-03-15 CRAN (R 3.4.4)
##  xfun          0.1       2018-01-22 CRAN (R 3.4.4)
##  xml2          1.2.0     2018-01-24 CRAN (R 3.4.4)
##  xtable        1.8-2     2016-02-05 CRAN (R 3.4.4)
##  yaml          2.1.18    2018-03-08 CRAN (R 3.4.4)
```
